# Isotope and archaeobotanical analysis reveal radical changes in mobility, diet and inequalities around 1500 BCE at the core of Europe

**DOI:** 10.1038/s41598-025-01113-z

**Published:** 2025-05-20

**Authors:** Claudio Cavazzuti, Anikó Horváth, Anett Gémes, Kristóf Fülöp, Tamás Szeniczey, János Gábor Tarbay, Ashley McCall, Beatriz Gamarra Rubio, Magdolna Vicze, Annamária Bárány, Ákos Pető, Enikő Katalin Magyari, Gabriella Darabos, István Futó, Zsuzsa Lisztes-Szabó, Erika Molnár, Mario Novak, Erika Gál, Klára P. Fischl, Gabriella Kulcsár, Vajk Szeverényi, Géza Szabó, Edit Mester, János Dani, László Palcsu, Viktória Kiss, István Major, Tamás Hajdu

**Affiliations:** 1 Department of History and Culture, Alma Mater Studiorum, University of Bologna, piazza San Giovanni in Monte 2, Bologna, 40124 Italy; 2 International Radiocarbon AMS Competence and Training Center (INTERACT), HUN-REN Institute for Nuclear Research, Bem tér 18/c, Debrecen, 4026 Hungary; 3 Department of Biological Anthropology, Institute of Biology, Faculty of Science, Eötvös Loránd University, Pázmány Péter sétány 1/C, 1117, Budapest, Hungary; 4 Salisbury Zrt. Tartsay Vilmos utca 14, Budapest, 1126 Hungary; 5 Institute of Archaeology, HUN-REN Research Centre for the Humanities, Tóth Kálmán u. 4, Budapest, 1097 Hungary; 6 National Institute of Archaeology, Hungarian National Museum Public Collection Centre, Múzeum krt. 14-16, Budapest, 1088 Hungary; 7 Independent researcher, Dublin, Ireland; 8 Independent researcher, Barcelona, Spain; 9 ELTE Museum of Natural History, Pázmány Péter sétány 1/c, Budapest, 1117 Hungary; 10 Department of Nature Conservation and Landscape Management, Institute for Wildlife Management and Nature Conservation, Hungarian University of Agriculture and Life Sciences, Páter Károly u. 1, Gödöllő, 2100 Hungary; 11 Department of Environmental and Landscape Geography, Institute of Geography and Earth Sciences, Center of Geography, Faculty of Science, Eötvös Loránd University, Pázmány Péter sétány 1/C, 1117, Budapest, Hungary; 12 Botanical Department, Faculty of Science and Technology, University of Debrecen, Egyetem tér 1, Debrecen, 4032 Hungary; 13 HUN-REN Institute for Nuclear Research, Bem tér 18/c, Debrecen, 4026 Hungary; 14 Department of Biological Anthropology, University of Szeged, Középfasor 52, Szeged, 6726 Hungary; 15 Centre for Applied Bioanthropology, Institute for Anthropological Research, Ljudevita Gaja 32, Zagreb, 10 000 Croatia; 16 Department of Archaeology, University of Miskolc, Building C1, Egyetemváros, 3515 Miskolc, Hungary; 17 Déri Museum, Déri tér 1, Debrecen, 4026 Hungary; 18 Wosinsky Mór Museum, Szent István tér 26, Szekszárd, 7100 Hungary; 19 Kiss Pál Museum, Tariczky sétány 8, Tiszafüred, 5350 Hungary; 20 Department of Archaeology, Faculty of Humanities, University of Szeged, Egyetem utca 2, Szeged, 6722 Hungary

**Keywords:** Bronze age, Diet, Mobility, Dental calculus, Panicum miliaceum, Anthropology, Archaeology

## Abstract

The transition from the Middle to the Late Bronze Age (around 1500 BCE) in the Carpathian Basin was parallel by drastic cultural changes in Central-Europe, which strongly influenced the dynamic of prehistoric Europe. The cultural fragmentation of the Middle Bronze Age (2000 − 1500 BCE) Carpathian Basin was followed by a more homogeneous development at the beginning of the Late Bronze Age (1500 − 1300 BCE), with the appearance of the Tumulus culture. In the beginning of this period, the long-used tell-settlements were abandoned, furthermore new pottery styles and metal types appeared. Whether these changes were caused by immigration, or a local adaptation to external influxes, has long been a matter of debate. Our study investigates this transition from the point of view of diet and mobility from several key-sites of Hungary. Our results show (1) low migration rates and a shift of migration trajectories; that (2) the beginning of the systematic consumption of *Panicum miliaceum* was from 1540 − 1480 BCE; that (3) the decrease of average animal protein intake was parallel by an increase of cereal consumption and a tendency to less unequal diet. Overall, our results shed new light on the dynamics of complex change in Bronze Age Europe.

**Supplementary Information:**

The online version contains supplementary material available at 10.1038/s41598-025-01113-z.

## Introduction

The Carpathian Basin was a core area in continental Europe regarding population dynamics, at least from the Early Neolithic (5900 BCE)^1^. In the Bronze Age (2600/2500 − 800 BCE), the region was subjected to migration flows from the eastern steppes^2^, and from the west during the Bell Beaker phase^3^. Evidence of newcomers has also been identified in the Early Bronze Age^4,5^as large-scale movements were not only associated with significant cultural changes but also had a substantial impact on the composition of the local Bronze Age societies^6^. These repeated flows of people were likely to be favoured by the available resources of the Great Hungarian Plain, with its abundance of waters, most notably of the Danube and Tisza rivers, the opportunities offered by diverse environmental niches, the fertile soils of the alluvial and loess plains, and the rich mineral and metal sources of the surrounding mountains.

Agriculture and husbandry represented the economic basis of the Bronze Age communities, together with intense craft production and long-distance exchange^7,8^. The participation to large-scale networks facilitated the spread of culture via the accumulation of different prestige goods, which in turn led to the increase of social hierarchies, well-reflected by the stratification of furnished graves, and the creation of fortified settlements, the so-called ‘tell settlements’^9,10^.

**Fig. 1:**
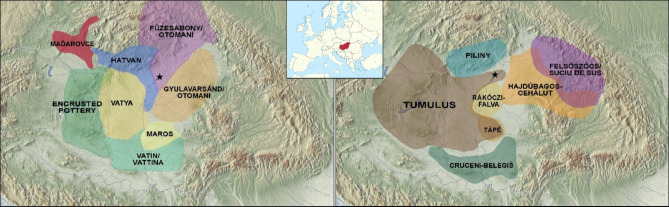
Geographic distribution of MBA (left) and LBA (right) cultures in the Carpathian Basin. The star symbol indicates the position of Tiszafüred-Majoroshalom cemetery. The map is constructed using “Natural Earth. Free vector and raster map data @ naturalearthdata.com” available at https://www.naturalearthdata.com/downloads/10m-raster-data/ and it was modified using QGIS open software freely downloadable https://qgis.org/. The distribution of the MBA cultural units are based on P. Fischl et al. 2013^12^, Fig. 2, while the distribution of the LBA cultural units are based on Kovács 1981^15^ Fig. 4 and research data of the authors.

The economic development that began in the Early Bronze Age (2600/2500 − 2000 BCE) with the dawn of bronze metallurgy was completed in the Middle Bronze Age (MBA) (2000 − 1500/1450 BCE) with the spread of tin bronzes^11^. By this time, we observe a complex pattern of different cultures (Fig. 1), characterized by specific settlement structures, material culture, and burial rites^12^. In the final phase of the MBA, known as Koszider Period (1600 − 1500/1450 BCE), however, the former regional differences leave space to a more unified picture. This process was fulfilled at the transition to the Late Bronze Age (LBA) with the appearance of the Tumulus Culture (TC) in several areas of the Carpathian, as well as in other parts of Central Europe^13^. With the appearance of the archaeological material of the TC, the 500-hundred-year-old tell settlement system along the Danube and in the Great Hungarian Plain were abandoned and the so-called ‘horizontal settlements’, with shorter periods of occupation, became widespread^14^.

Whether the communities that were associated with the TC were local, which would indicate genetic continuity, or originated from episodes of migration-admixture is still a matter of debate among archaeologists^15,16^,^17^. An archaeological^18^ and a craniometric study^19^showed discrepancies between MBA and LBA groups in Tiszafüred, in contrast to the previous suggestions^20^. In this sense, the Carpathian Basin represents a unique geographical context to explore the dynamics of cultural and territorial shifts in prehistory, by analysing the mobility patterns, as well as changes in diet and lifestyle of those living in and migrating through the area.

**Fig. 2:**
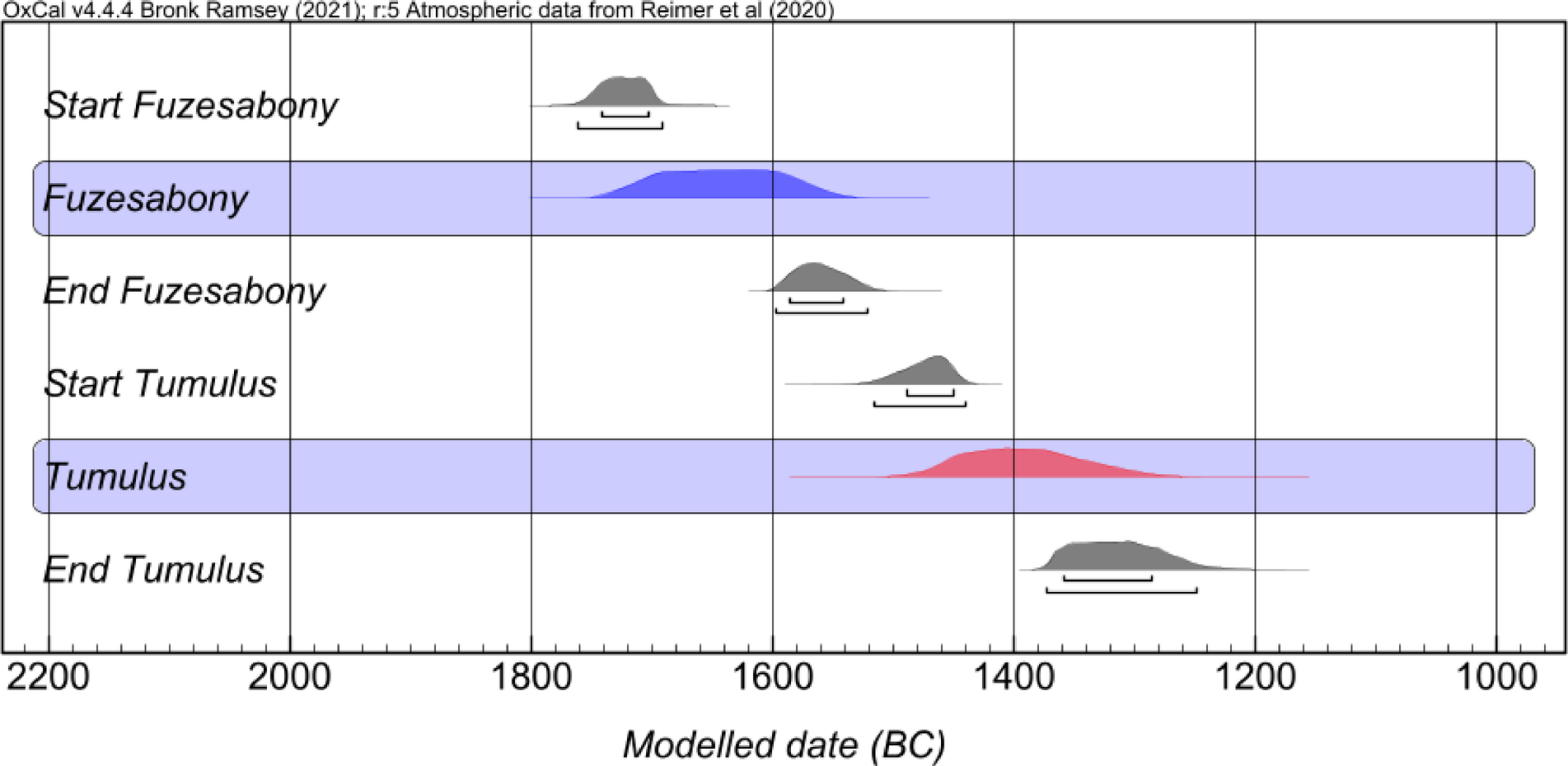
Cumulative radiocarbon curve for MBA (Füzesabony culture) and LBA (Tumulus culture) individuals from Tiszafüred-Majoroshalom cemetery (data from: 21).

Here we present 130 new isotope data (δ^13^C, δ^15^N, ^87^Sr/^86^Sr) obtained from 46 human and 10 animal samples found at Tiszafüred-Majoroshalom cemetery, complemented by other 14 human samples from key-contexts located in the Upper Tisza River basin (Gelej-Kanálisdűlő, Csanytelek-Palé, Tiszapalkonya-Erőmű, Rákóczifalva-Kastélydomb) dated to the MBA (Füzesabony and Vatya cultures) and the LBA (Tumulus culture, TC) (Table S1). Through the use of a large number of radiocarbon dates^21^, we reached an unprecedented chronological resolution, which allowed us to restrict substantially the timing at which change occur (Fig. 2; Table S2).

## Results

### Mobility: strontium isotope analysis

The five analysed burial sites are located in the upper and central basin of the Tisza River, which originates in the Carpathian Mountains and flows southward crossing the Great Hungarian Plain. The major site, Tiszafüred-Majoroshalom, as well as Tiszapalkonya-Erőmű, Rákóczifalva-Kastélydomb, and Csanytelek-Palé are situated on the Tisza riverside, while Gelej-Kanálisdűlő is around 20 km west of the present-day riverbed. Due to the overall geolithological and geomorphological uniformity of the plain in this region, the bioavailable strontium baselines are homogenous, with ^87^Sr/^86^Sr ranging from 0.7091 to 0.7101 (Fig. S1)^22–25^,^26^. More radiogenic values (0.7101–0.7111) are observed in the volcanic formations of the Bükk Mountains and in the Upper Tisza basin, closer to the Carpathians. Further east, in Romanian territory, among the volcanic and sedimentary formations of the Eastern Carpathians, less radiogenic values are documented (< 0.7080)^27–29^.

The distribution of human ^87^Sr/^86^Sr values highlights the potential territorial variability of baselines in a region of around 150 km along the Tisza River basin (Fig. S2). At Tiszapalkonya-Erőmű and Gelej-Kanálisdűlő values are more radiogenic compared to Tiszafüred-Majoroshalom and Rákóczifalva-Kastélydomb, possibly as consequence of their proximity to the northern mid-mountain region. Csanytelek-Palé, the southernmost site, shows the less radiogenic values. The Tisza basin, despite the apparent geolithological homogeneity, therefore, exhibits slight but significant discrepancies of ^87^Sr/^86^Sr composition. Despite our primary focus on Tiszafüred-Majoroshalom cemetery, this overview highlights the potential mobility between different sub- and micro-regions.

**Fig. 3:**
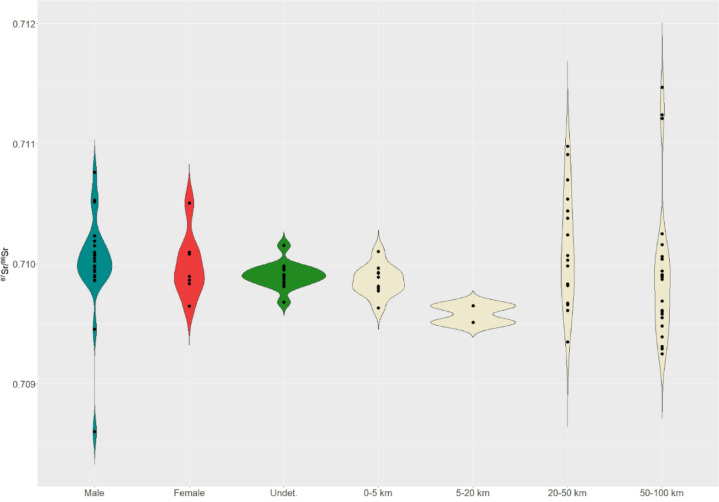
Comparison between human and territorial ^87^Sr/^86^Sr data distributions. Humans are subdivided into adult males, adult females, and undetermined sex individuals, most of which are subadults. Baselines are subdivided into areal categories at different radii from Tiszafüred-Majoroshalom site.

After a general overview of the regional isoscape, we drew a more detailed picture of the Tiszafüred-Majoroshalom area. We collected and analysed ^87^Sr/^86^Sr baselines at different distances from the site (Table S3), in order to model mobility at a territorial scale (Fig. S3), following several examples from the literature^26,27^,^28^. Looking at the Tiszafüred-Majoroshalom territory, we observe a restricted range of ^87^Sr/^86^Sr values within 20 km from the site (0.7095–0.7101). At 20–50 km radius, the baselines range between 0.7094 and 0.7110, while at a 50–100 km radius they vary between 0.7093 and 0.7115 (Fig. 3).

The distribution shows that 35 of the Tiszafüred-Majoroshalom are compatible with 0–20 km range and are, therefore, potentially indigenous or from the immediate hinterland. Ten individuals show more diverse values, instead compatible with the broader hinterland baselines (within 50 km), and only one adult male fall unambiguously outside the local and hinterland range. All infants, except one, appear indigenous. Adult males seem slightly more mobile than females, but the broader amplitude of male isotope variability is probably due their higher samples size. Hence, considering all the caveats of strontium isotope analysis, Tiszafüred-Majoroshalom’s population seems mostly local, with a relatively intense mobility within the hinterland, and at least few immigrants from more remote regions.

**Fig. 4:**
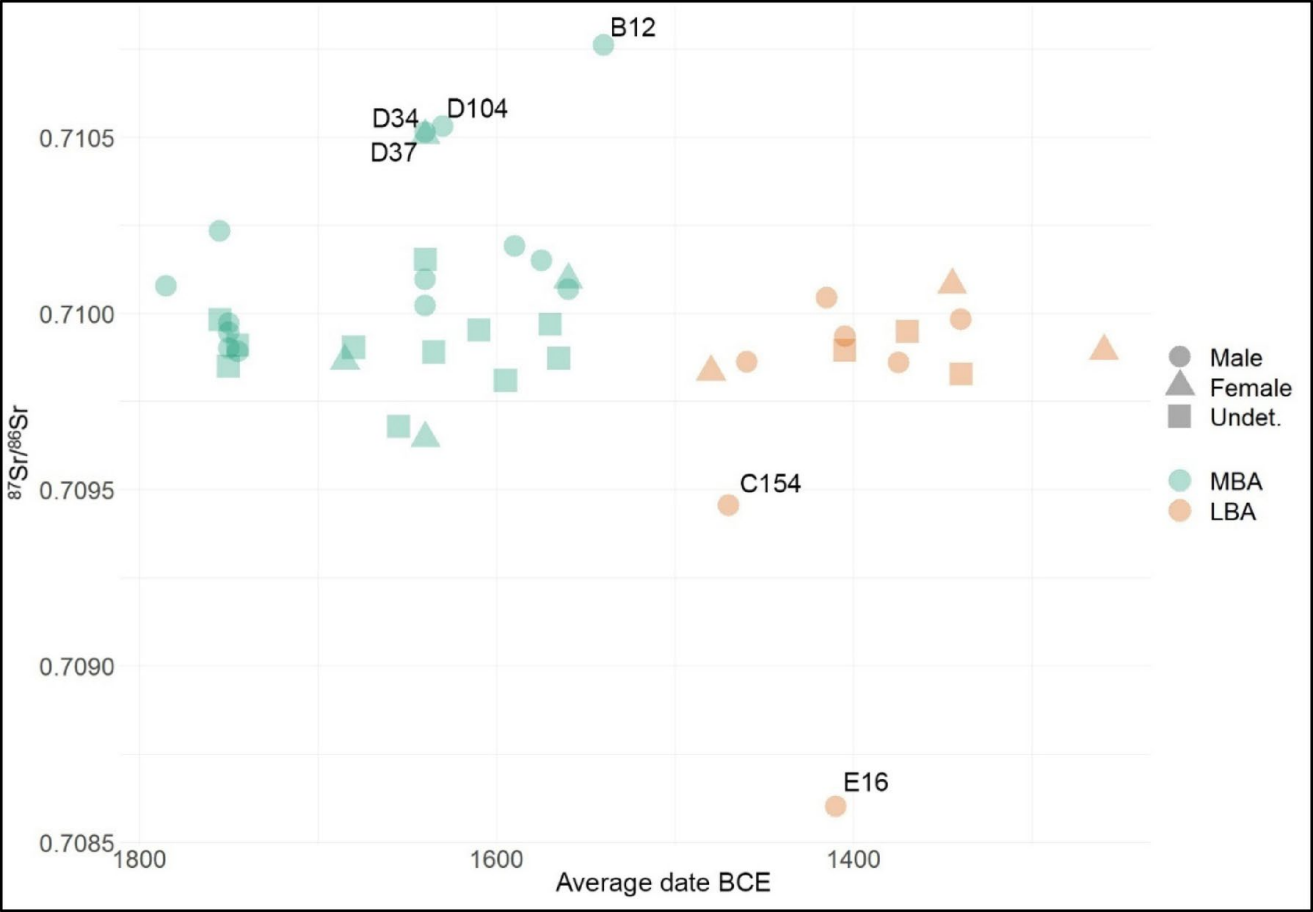
^87^Sr/^86^Sr and^14^C data from Tiszafüred-Majoroshalom cemetery subdivided per estimated sex and chronological phase. The codes indicate burials of the most evident outliers^14^C data are provided in (21).

Integrating ^87^Sr/^86^Sr results with radiocarbon dates, we observe changes of mobility pattern through time (Fig. 4). In general, the variability of more local individuals slightly tends to restrict at the transition to the LBA, possibly as a consequence of less frequent mobility throughout the hinterland. Considering the similar trend of carbon and nitrogen isotope values, which will be shown below, this might also be explained with the use of more local/less various foods by the LBA community. Based on the principle of strontium isotope composition, we cannot exclude more remote provenances in both periods from regions that are characterized by similar geolithology.

Notably, while the origins of MBA immigrants (D34, D37, D104, B12) point to more radiogenic areas (e.g. Upper Tisza basin, or Northern Carpathians), the LBA outliers (E16 and C154) seem to come from less radiogenic areas, such as the Middle Danube, the Southern Carpathians, or Transdanubian hills. These data are of particular interest for the dynamics that determined the appearance of the TC in the Great Hungarian plain, which we will discuss in the following paragraphs.

### Diet: carbon and nitrogen isotope analysis

**Fig. 5:**
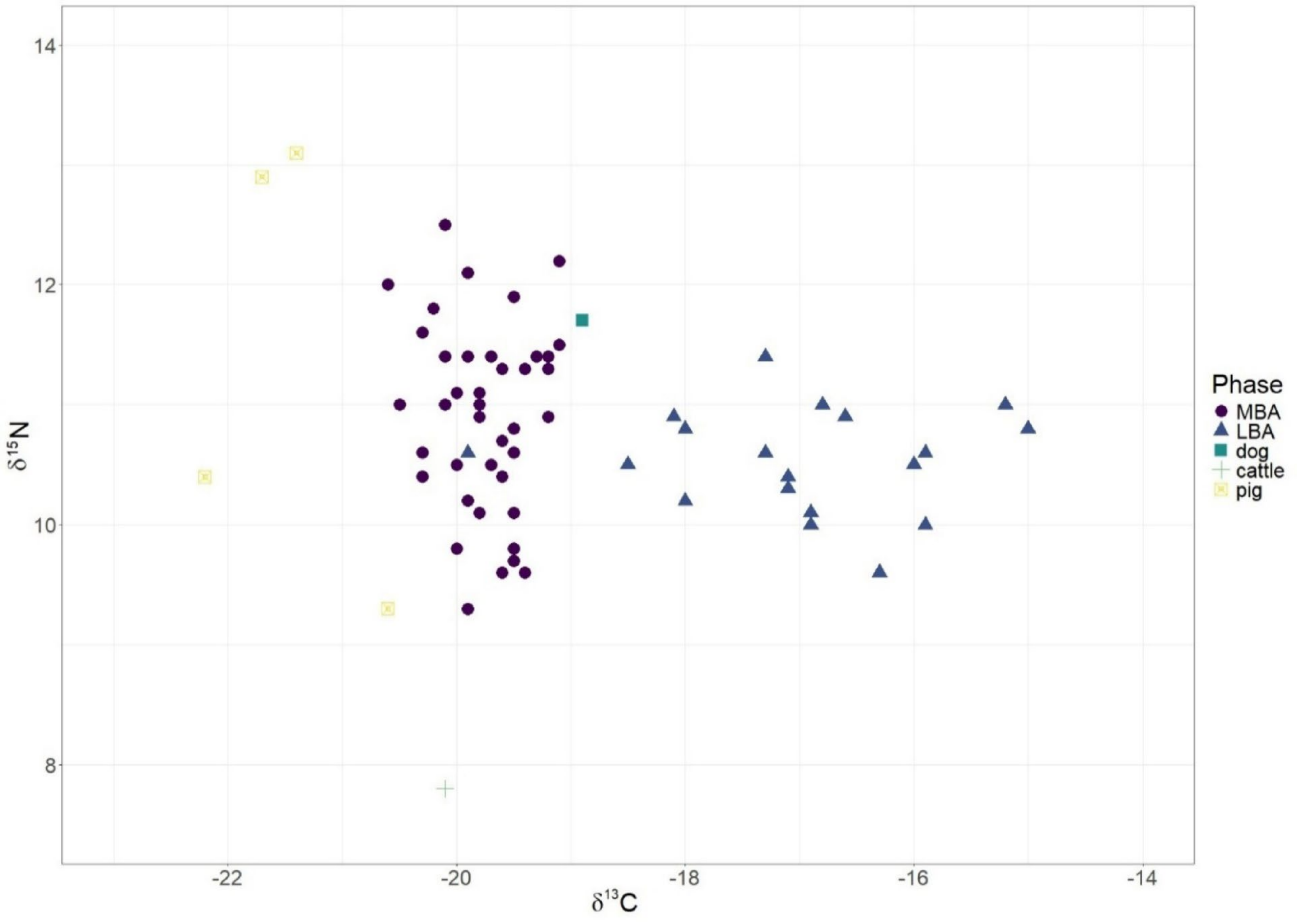
Human and animal δ^13^C and δ^15^N data across the MBA and LBA.

We analysed the collagen extracted from 62 human samples from the five sites, 44 of which were from Tiszafüred-Majoroshalom cemetery and 6 animal samples from Tiszafüred-Majoroshalom tell settlement, which was abandoned at the end of the MBA. (Fig. 5). Therefore, animal remains are dated to the MBA phase.

The comparison between fauna and human medians (δ^13^C_humans−fauna_=−19.5‰ - −20.6‰ =1.1‰; δ^15^N_humans−fauna_=10.8‰ - −10.7‰ =0.1‰) shows predictable results regarding carbon, and, by contrast, an unexpected small difference in nitrogen. Indeed, the diet-tissue trophic enrichment factor is estimated to range between 0 and 1‰ for the carbon and between 3 and 5‰ for the nitrogen per trophic level^29,30^. The slight discrepancy in nitrogen medians is due to the presence of two pigs and a dog with high δ^15^N values, 13.1‰, 12.9‰, and 11.7‰, respectively. This could be the result of being partially fed with the remains of human meals, comprising a significant quantity of proteins. If we consider herbivores exclusively, then δ^15^N_humans−fauna_=10.8‰ −7.8‰ =3‰, which appears more coherent with the expected trophic level variation. Interestingly, pigs show δ^13^C values ranging between − 20.6 and − 22.2‰, which indicates the absence of C4 plants in their diet.

Concerning humans, the isotopic values indicate a mixed terrestrial diet, with possible addition of riverine fish, in some cases. The mean values during the MBA are typical of a C3-plant based with a highly variable protein intake, probably due to a differential access to protein resources in this phase. However, two clear tendencies emerge at the transition to the LBA.

**Fig. 6:**
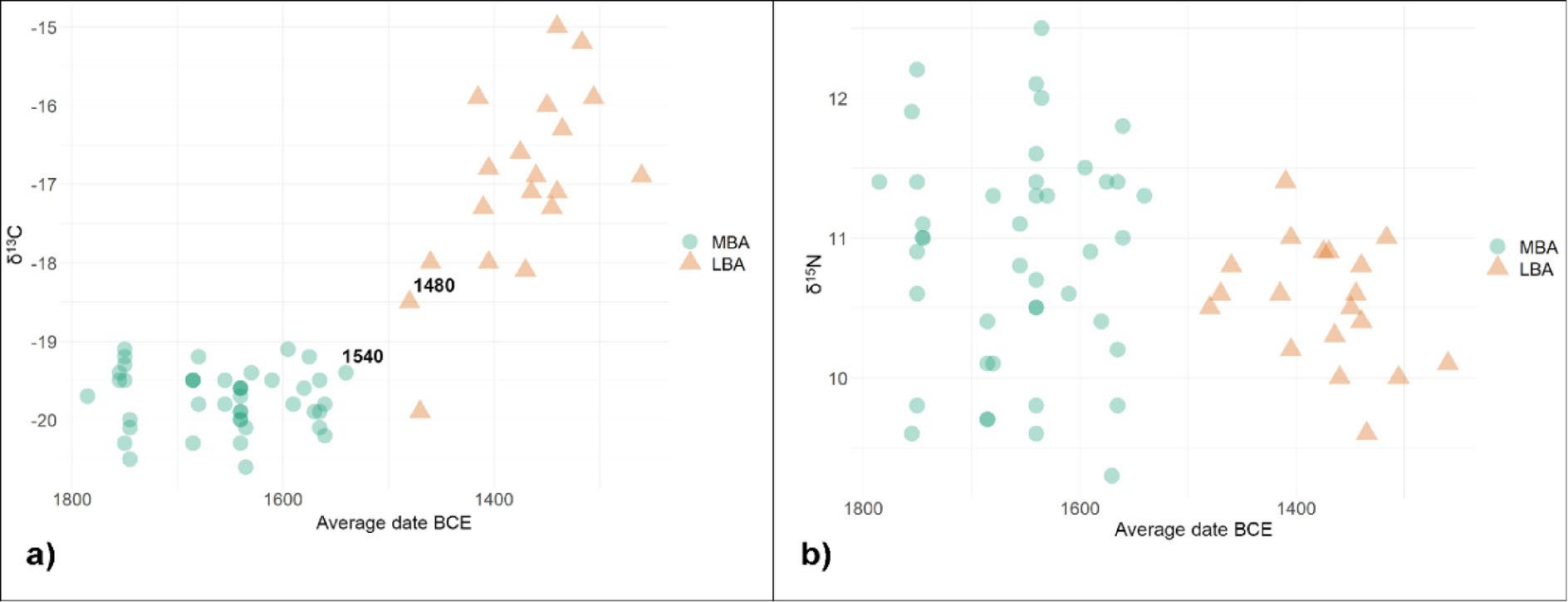
Variability of human δ^13^C (**a**) and δ^15^N (**b**) data over time. 14C dates (cal. BCE) of the latest MBA and the earliest LBA individuals to emphasize the time range of dietary shift towards systematic consumption of C4 plants.

First, we observe a significant shift towards higher δ^13^C values, which we interpret as a consequence of the introduction and progressive increment in time of C4 plants in the human diet, most notably broomcorn millet (*Panicum miliaceum *L.)^31–33^. Radiocarbon dates indicate that the dietary change occurred between 1540 and 1480 cal. BCE (Fig. 6a). One adult male from Tiszafüred-Majoroshalom cemetery (id: C154), despite his dating to the LBA Tumulus Culture, still shows a typical C3-plant-based diet. C154, moreover, exhibits a^87^Sr/^86^Sr value that is slightly less radiogenic of the 0–20 km range, pointing to an origin from the hinterland or another more remote region with a similar geolithology. Other four individuals dated between 1480 and 1370 BCE with δ^13^C values proximal to −18‰ also show a limited consumption of C4 plants.

The second clear change is the drastic reduction of the variability of δ^15^N values at the transition to the LBA (Fig. 6b). A possible explanation of this phenomenon might be a reduction of food variety and/or a restriction of socio-economic inequalities, which may be reflected by more marked differences in the access to animal protein resources.

We also tested the existence of differential access to proteins between adults of different sex, estimated through osteological analysis. During the MBA the differences are statistically significant (t = 3.1444, df = 28, p-value = 0.003918), with males showing a tendency towards more protein intake, while during the LBA the two distributions do not vary significantly (t = 1.1572, df = 11, p-value = 0.2717) (Table S4).

### Diet: dental calculus (micro-archaeobotany)

With the dental calculus micro-remain analysis, we focused on answering the question of whether the increased δ13C values observed in LBA individuals could have been caused by a more systematic consumption of C4 cereals, most notably broomcorn millet. Dental calculus samples were collected from 25 individuals from Tiszafüred-Majoroshalom and micro-remains were detected in 80% of the sample set (20 out of 25). Since the examined individuals were predominantly adult males, we cannot make statistical inferences about potential age or sex differentiated diets.

**Fig. 7:**
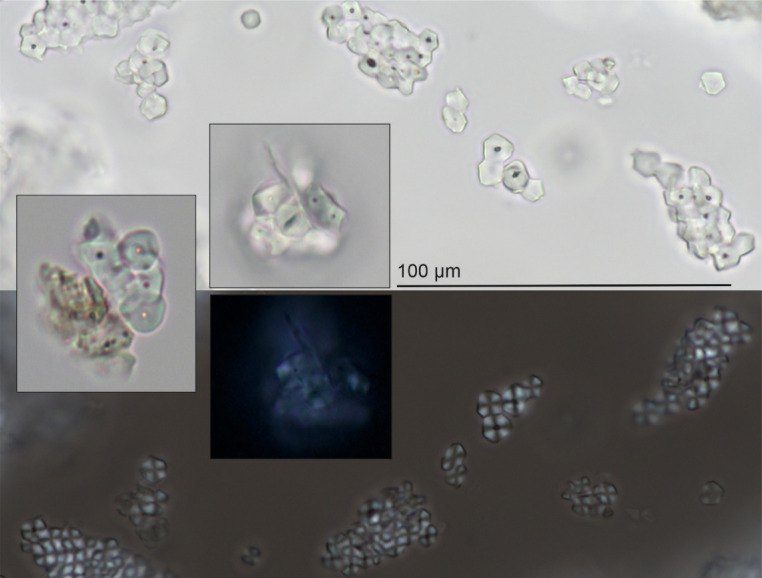
Light and polarized microscope images of millet starch granules from modern reference samples in the background and from LBA dental calculus samples in the embedded pictures.

Dental deposits contained starch granules, fibers, phytoliths, micro-charcoal, pollen, animal and plant egg cells and spores, fungal remains, and unidentified plant and animal remains (Fig. 7). Micro-remains related to cereal grains were starch granules with clear characteristic details for barley (*Hordeum* sp.), wheat (*Triticum* sp.), and broomcorn millet, in addition to transversal cells of the pericarp of the cereal grains, fibers from crop grains, and phytoliths or silica skeletons. Gelatinized starch remains were not involved in this analysis because it cannot be excluded that these are from legume plant starch.

Triticeae and millet starch grains were distinguishable based on their size, and shape and based on whether they appeared in groups or alone. The transversal cells of the cereal grain pericarp could not be identified at the species level, similar to the fibers from crop grains. Some phytoliths embedded in dental plaque had taxonomical relevance. Phytoliths of long cells in the chaff (lemmas, paleas, and glumes) of cereal grains mostly refer to Triticaea plants in our samples. The shape of the found short cell phytolith (Bilobate) refers to broomcorn millet. In addition, there were silica skeletons in the dental calculus that could not be identified by taxon. We found silicified remains (phytolith, silica skeleton) for one MBA individual and four LBA individuals.

**Fig. 8:**
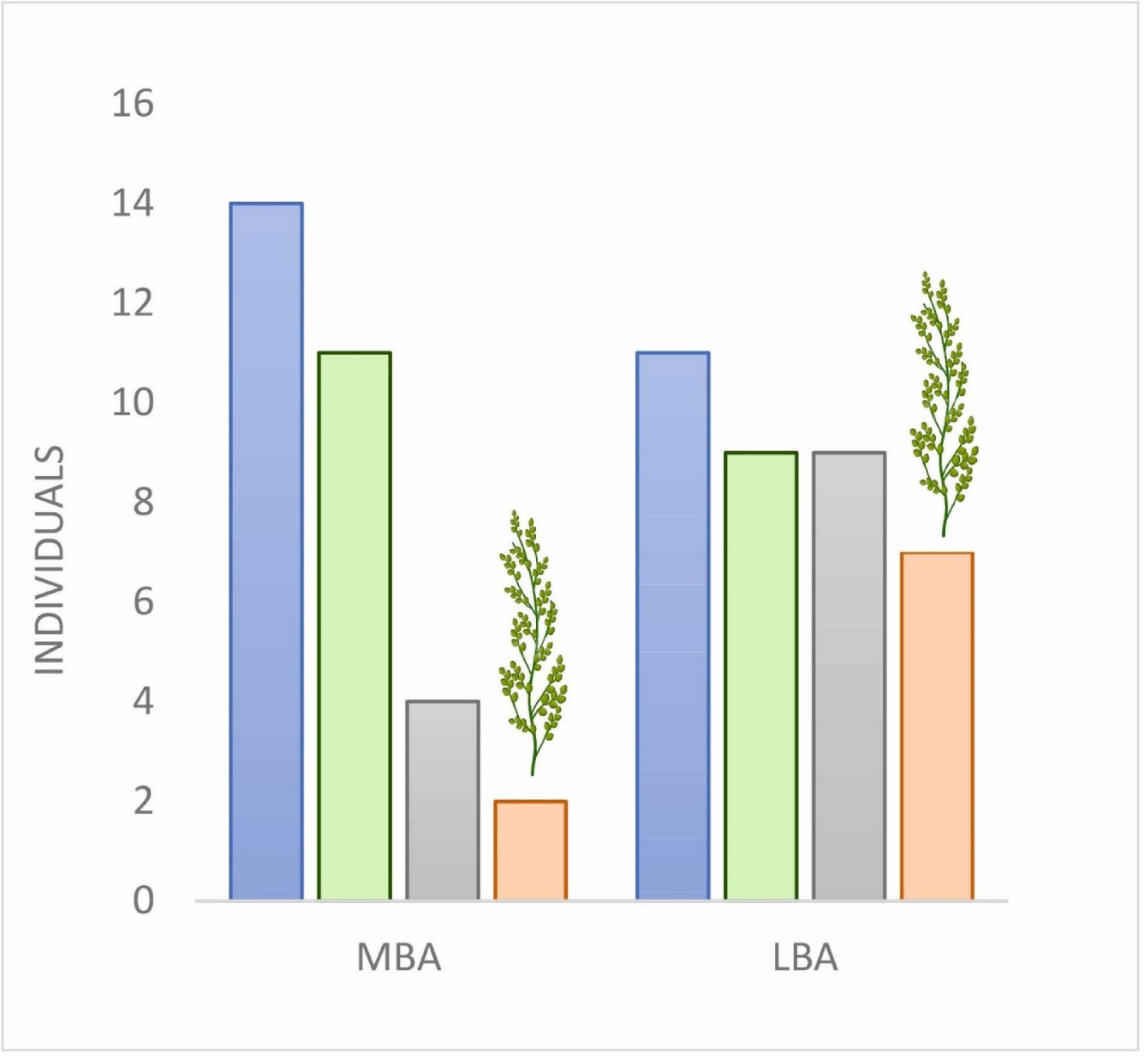
Summary of the dental calculus analysis results for the Tiszafüred-Majoroshalom site. Blue bar: sampled individuals; green bar: individuals with dental calculus containing microremains; grey bar: individuals with cereal microremains; orange bar: individuals with broomcorn millet microremains.

Traces of cereals were present in 11 out of the 14 MBA subjects, while the frequency increases in LBA (Fig. 8). Among the 11 LBA subjects, in 9 cases, traces of cereals could be detected. In the MBA, the dental calculus of only two individuals from the 14 (tombs n. D37 dated 1740 − 1540 cal. BCE and B69 dated 1620 − 1500 cal. BCE, 2σ) contained broomcorn millet starch, while in the LBA period the frequency raises 6 from 11.

### Diet: macro-archaeobotany

In order to complement the direct evidence of subsistence, choices provided by the dental calculus analysis, 22 sediment samples were collected from the MBA context of Tiszafüred-Majoroshalom (Feature No. 4.) and processed through the standard archaeobotanical protocol. From the quantitative point of view the retrieved carpological record of the samples is scarce. It is important to note that remains of broomcorn millet could not be identified. Only samples 4/135 yielded a single, fragmented carpological remain, which might be identified as caryopsis of *Setaria*/*Panicum*. Samples provided carpological remains of wheat were detected in almost 60% of the samples (13/22), while barley were found in less than 10% (2/22). Still, unidentified cereal caryopses fragments of non-Panicum cereals were detected in almost all samples (21/22). Besides barley, remains of emmer and spelt were found; moreover, no signs of einkorn were discovered. While LBA phases are well-documented in the cemetery, the Tiszafüred-Majoroshalom tell shows no LBA layers, due either to the abandonment of the site around 1500 BCE or to the modern agricultural disturbance, we could not verify the eventual presence of broomcorn millet in the LBA. However, the absence of preserved broomcorn millet macro-remains in the MBA layers seems to confirm the scarce presence of this cereal prior to 1540 and its introduction around 1480 BCE, as suggested by carbon isotope and dental calculus analysis.

## Discussion

^87^Sr/^86^Sr data in the other four sites reveal slight but significant discrepancies across the various subregions of the Tisza basin, which appears promising for future mobility studies at a regional and sub-regional scale. Tiszafüred-Majoroshalom burial site provides a sufficiently large sample size for discussing human mobility patterns. Overall, the majority of the analysed individuals appear indigenous or from the immediate hinterland (0–20 km), approximately one-fifth is compatible with the broader hinterland (20–100 km), and only one can unambiguously be identified as an immigrant from a distant region (> 100 km). Due to the low degree of geolithological/isotopic variation in the Great Hungarian Plain, however, we cannot exclude that some of the subjects whose ^87^Sr/^86^Sr ratio is compatible with the local baselines might come from different regions characterized by similar geolithological spectrum and, therefore, are of coincidentally similar isotopic composition.

Mobility is more intense among adult males, although this might depend on their greater number among the sampled individuals. Female exogamy does not appear very frequent, and if present, it was probably practiced within the hinterland. The focus on MBA-LBA transition reveals a trend towards a reduction of strontium isotope variability, as well as a change of migration trajectories.

While the origins of MBA immigrants point to more radiogenic areas (e.g. Upper Tisza basin, or Northern Carpathians), the LBA outliers seem to come from less radiogenic areas, such as the Middle Danube, the Southern Carpathians, or Transdanubian hills, namely the regions of expansion of the TC towards east. The early dates of the two LBA outliers (1500 − 1400 BCE) seems to support the idea of a certain degree of migration from the west towards the Great Hungarian Plain at the onset of the TC, which would corroborate previous hypothesis, based on the trajectory of diffusion of material culture^15–18^, and bioanthropological data^19^.

Diet indeed provides major evidence of drastic change between the two phases. δ^15^N data distribution indicates a substantial reduction of average protein intake, which seems to contradict the old *topos *of the “pastoralist Tumulus Grave Culture”, investing primarily on husbandry and animal food resources^34^. Moreover, the wider variability of nitrogen isotope values during MBA suggests greater food variety and a more differentiated access to protein in this phase. MBA society was probably more stratified: the existence of established social hierarchies could imply strong inequalities in the access to foods of animal origin, especially within the male component. The abandonment of the fortified MBA tell settlement of Tiszafüred-Majoroshalom, which coincides with the broader collapse of the political system in the region, is followed by a new phase with less structured centres, less stable social hierarchies and the reduced inequalities that we could clearly observe, at least at the level of food consumption.

Besides that, we observe the increasing proportion of C4 plants consumption in human subsistence among individuals ascribed to the Tumulus culture (LBA), with the above-mentioned only exception of grave C154. The earliest burial that shows a significant consumption of C4 plants is dated to 1530 − 1430 BCE, in conjunction with the appearance of the broomcorn millet around 1500 BCE^32^. According to our results, the strong investment in broomcorn millet as key-cereal in the Carpathian Basin’s community started at some point between 1540 and 1480 BCE, which is considered one of the earliest phases in Europe^31^,^32^,^35–37^. One of the most ancient macro-remain finds of broomcorn millet so far comes from the site of Fajsz in southern Hungary, which has also been directly raiocarbon dated to the period between 1600 and 1400 BCE^38^. With our study, we confirmed and added chronological resolution to this significant evidence.

Our stable isotope results are corroborated by microscopic analysis of the dental calculus of the individuals from Tiszafüred-Majoroshalom. Cereal consumption was more relevant to the diet of LBA than in MBA individuals. In the MBA, two-thirds of the analysed individuals did not provide remains of cereals; the remaining specimens showed traces of wheat and barley-related carpological remains. The observation gained through the micro-archaeobotanical analysis might be another element to support that diet was more varied in the MBA than in the LBA.

During the MBA, a wide range of cultivated cereals was already available in Europe, e.g. barley (*Hordeum vulgare* L.), einkorn (*Triticum monococcum* ssp. *monococcum* L.), emmer (*Triticum* turgidum ssp. *dicoccum* (Schrank.) Thell.), spelt (*Triticum aestivum L.* ssp. *spelta* L. Thell.), and other hexaploid and tetraploid types of wheat (*Triticum aestivum*species)^39,40^. Although barley and einkorn were the main cereals, the MBA showed a flourishing in terms of the diversity of cultivated plants, among the archaeobotanical finds were legumes (Fabaceae), such as lentils (*Lens culinaris* L. Medik.), ervil or bitter vetch (*Vicia ervilia* L.), oil and fiber crops such as safflower (*Carthamus tinctorius* L.) and flax (*Linum usitatissimum*L.)^41^. Moreover, there is ample evidence of human consumption of collected plants^41,42^,^43^.

Based on our dental calculus analysis, the proportion of consumed cereal increased at the beginning of the LBA. Moreover, although there were already few but evident signs of broomcorn millet consumption in the latest phases of the MBA (tombs n. D37 dated 1740 − 1540 cal. BCE and B69 dated 1620 − 1500 cal. BCE, 2σ), almost all of the LBA cereal-consuming individuals showed signs of millet consumption at the same time (7 out of 9 individuals). We can conclude that broomcorn millet must have become a characteristic food of the LBA period, as other archaeobotanical data confirm^37,39,44,45^. This may be due to its high degree of resistance, high potential of productivity and short maturation period. Its cultivation could contribute substantially to increase the carrying capacity of LBA village communities and support demographic pressure in times of growing demand of food resources.

Together with spelt and emmer, millet eventually might have prevailed over einkorn and barley. Similarly, the consumption of foxtail millet as a plant causing carbon enrichment may arise, but according to Zohary et al.^46^, the first evidence can only be detected in Europe after 1200 BCE. It should be noted, hovewer, that in many cases the identification of *Setaria* and *Panicum *species can be difficult. Based on the representativeness indices of Stika and Heiss^47^, Effenberger^48^ presented an estimated cereal consumption summary for the LBA of the Carpathian Basin region, with broomcorn millet slightly below 20%, and scarce traces of foxtail millet. Whether foxtail millet was present as a weed, was grown on its own, or it was co-produced with broomcorn millet still has to be clarified.

In conclusion, our dataset confirms that the change of material culture, settlement patterns, and burial customs that occurred in the Carpathian Basin in concomitance with the appearance of the TC (1500 BCE) also coincided with a substantial modification of other fundamental aspects of culture, namely subsistence practices, primary economy, dietary habits, and presumably, cuisine. The change of mobility trajectories we could detect requires an extensionof isotope data to obtain confirmation and, possibly, achieve a more general picture of the historical phenomenon. Future aDNA data may provide further evidence in terms of migration and social permeability, although other social, political and geopolitical phenomena, including intra- and inter-community conflicts, might explain such an extraordinarily dynamic epochal transition.

## Materials and methods

### Materials

The investigated MBA and LBA human remains are curated at the Hungarian Natural History Museum, Budapest (Tiszafüred-Majoroshalom, Tiszapalkonya-Erőmű and Gelej-Kanálisdűlő), Kiss Pál Museum, Tiszafüred (Tiszafüred-Majoroshalom III) and Department of Biological Anthropology at Szeged University (Csanytelek-Palé and Rákóczifalva-Kastélydomb). The main information about the archaeological contexts, as well as the number of analysed specimens, is provided in the Supplementary text and Table S1.

### Methods: isotope analysis

Stable isotope analysis is of primary importance in bioarchaeological studies, not only in tracing past subsistence strategies and mobility patterns, but also in reconstructing environmental and living conditions and past migration routes^49,50^. Isotopes from food and drink become incorporated in the individual’s bone collagen and bioapatite in the teeth, the Sr isotope composition of which thus reflect the geology of the place of residence at the time of tissue formation or remodelling. The approach relies upon variations in the Sr isotope composition of rocks of different ages and lithologies, reflected by associated soils and the bioavailable Sr (that is available to plants and thus, enters the food chain). Sr isotope ratio (^87^Sr/^86^Sr) in tooth enamel represents various short periods of childhood and adolescence (depending on which tooth it comes from) and is highly resistant to post-mortem diagenesis. In summary, human core enamel and bones can, under favourable conditions, be used to reconstruct mobility patterns from childhood to later life^23^. For a relevant archaeological evaluation of isotopic data, one must consider the species, lifestyle, diet, probable extension of living space, and age at death (cell turnover rate) of the individual. For non-local populations, it might be possible to determine a geographical area of origin based on a Sr isotope ratio map.

Understanding diet is one of the key aspects to assess the socio-cultural and technological differences between and within human groups. The analysis of carbon and nitrogen isotope ratios (expressed as δ^13^C and δ^15^N) from the collagen of human skeletal elements enables investigating variations in dietary behaviours of past human populations. The δ^13^C and δ^15^N isotopic composition of collagen provides an indication for the sources of the proteins consumed by individuals in the last ~ 10 years of their life^51,52^. To obtain reliable estimations for humans’ diet, the information from the isotope compositions of lower levels of the food web (baseline δ^13^C and δ^15^N), which are linked to and influenced by the environmental factors (e.g., climate, soil, humidity, canopy effect, etc.) have to be taken into consideration^53^. In the Carpathian Basin, dominated by C3 plants, the elevation in δ^13^C indicate the consumption of marine/aquatic food or C4 plants, while its decline can be tracked back to canopy effects of closed forests. The δ^15^N value can be used to distinguish between organisms from different trophic levels within a food web^30^. In addition, the δ^15^N value indicate the fraction of animal and plant-derived protein in human diets, in which aquatic foods have generally higher δ^15^N values compare to the terrestrial foods. Terrestrial animals get their nitrogen from plants, and plant δ^15^N are influenced by the nutrient inputs (manure or mineral fertilizers), extend of nitrification and denitrification processes, temperature, humidity, and pH of the soil.

Sr, C, and N isotope analyses were performed on human bones and teeth from five Bronze Age sites located in the Tisza basin, namely Tiszapalkonya-Erőmű and Gelej-Kanálisdűlő (Füzesabony culture, MBA), Csanytelek-Palé (Vatya culture, MBA), Rákóczifalva-Kastélydomb (Rákóczifalva group of the Tumulus culture, LBA), and Tiszafüred-Majoroshalom (Füzesabony and Tumulus culture cemeteries, MBA and LBA) which covers the entire focus period between MBA and LBA between 1800 − 1300 BCE^56^. Overall, the number of carbon/nitrogen and strontium isotopic ratios were obtained from 58 bone and 60 canine, first or second molar enamel samples (Table S1). The full list of sampled burials with specific indications of chronology, culture, estimated sex/age-at-death, as well as isotope data are reported in Supplementary Dataset 1 and Table S2. The detailed archaeological description of the chosen graves and the results of the radiocarbon analysis of the human remains of the investigated graves have recently been published^21^.

Regarding ^87^Sr/^86^Sr analysis, tooth enamel samples were cut and cleaned to remove dentine at the International Radiocarbon AMS Competence and Training Center (INTERACT), Institute for Nuclear Research, Debrecen. In order to remove surface contaminants prior to cutting, the surface was abraded with a diamond tipped Dremel 4000 tool. Any remaining dentine affixed to the enamel was removed using a diamond tipped Dremel 4000 tool and checked under a stereo microscope. All sample preparation occurred in a Class 1000 cleanroom. Between 20 and 40 mg of cleaned enamel samples were weighed and placed into labelled PFA beakers. Additional blank and standard solutions were included to verify the blank and accuracy of the chemical preparation. Enamel samples were digested twice in 1 mL of 14 M HNO_3_ and placed on a hotplate at 120 °C to evaporate to dryness. When the samples were fully demineralized, they were taken up in 8 M HNO_3_ and loaded into the precleaned Sr spec column to separate Sr from the other matrix elements. Crown ether-based Sr-Spec Resin (50–100 μm particle size) from Triskem International, France was used. Polypropylene 2 ml columns were filled with the resin and used as a chromatographic column. Polyethylene frit material was set on the top of the resin. Resin was pre-cleaned with 8 M HNO_3_ and UPW prior to use. Sr was isolated from the matrix components and from Rb to avoid an isobaric overlap of ^87^Sr^+^ and ^87^Rb^+^. Following this separation, the solution was evaporated to dryness, and 1 mL of 14 mol L − 1 nitric acid was added, and the sample was evaporated again. The residue was taken up in 4 mL 3% HNO3 solution. Standard solutions were prepared from the NBS987 SrCO_3_. Strontium isotope ratios were measured using a Neptune Plus MC-ICP-MS (ThermoScientifc), equipped with an Aridus-3 (CETAC) system. The ^87^Sr/^86^Sr ratio was corrected for instrumental mass discrimination using ^88^Sr/^86^Sr=8.375209, as well as by applying an interference correction for ^87^Rb^+^ and ^86^Kr^+^ with ^85^Rb^+^ and ^83^Kr^+^, respectively. All values were normalized to the accepted value of 0.710248 for NBS987^54^. Results obtained from human enamel analysis were then compared to the available baseline data from environmental sources^22^, from which we then developed an updated ^87^Sr/^86^Sr isoscape for the Carpathian Basin.

As regards to δ^13^C and δ^15^N, collagen extraction was also performed at INTERACT following a modified version of the method by Longin^55^. Each sample was weighed to ~ 0.3 mg and placed into an ultraclean aluminium capsule. All samples were combusted and measured three times to assure repeatability, i.e. within the acceptable range of two standard deviations of each other. Stable carbon and nitrogen isotope results were obtained by a Thermo Finnigan Delta^Plus^XP isotope ratio mass spectrometer, coupled with an elemental analyzer interface (Thermo Scientific™, EA IsoLink CNSOH). Measuring the collagen material and international isotope standards (IAEA-N1, IAEA-N2, IAEA-600, UREA) repeatedly in the same run, the stable carbon and nitrogen isotope ratios were determined with an overall uncertainty of ± 0.1‰, respectively^56^. All carbon stable isotopic results are expressed as a delta (δ) value relative to Vienna Pee Dee Belemnite (VPDB), and all nitrogen stable isotopic results as a delta (δ) value relative to ambient air (AIR).

Samples were assessed for contamination based on carbon and nitrogen content or weight (%). Acceptable %C ranges for modern mammalian bone collagen are between 15.3% and 47%, and for %N between 5.5% and 17.3%; samples falling outside those ranges were deemed inappropriate for analysis.

### Methods: dental calculus micro-remain analysis

To retrieve information from the plant micro-remains in dental calculi, the sampling procedure was conducted in the microfossil laboratory of the Institute for Nuclear Research where food or drink was never allowed to prevent sample contamination. During the dental calculus sample preparation procedure, powder-free gloves were worn in starch-free conditions. First, the teeth were visually examined. The teeth in the jaw and the spaces between the teeth were washed with ultra-pure water (UPW) to remove the post-mortem coarse contamination. After thoroughly brushing with an original soft bristle toothbrush using UPW, teeth were rinsed with running UPW. The toothbrush was rinsed with 99% ethyl alcohol and UPW between uses to avoid cross-contamination. After air drying, the dental calculus was carefully removed with a dental curette. In the case of considerably dust-covered skeletons, where the cleaning would have been complicated in this way, we separated large pieces of connected dental plaque from the teeth. These dental plaques were rinsed carefully two or three times with ultrapure water in Eppendorf tubes, and then the plaques were picked up with tweezers and left to dry on filter paper.

The samples were processed based on King et al.^57^. Retrieved calculus fragments were placed in 2 ml Eppendorf tubes, and 1 ml 10% hydrochloric acid was added to dissolve the matrix of the calculus fragments and release plant microfossils. Stirring in acid solution lasted until no more calculus fragments were visible (5 min to 1 h), and samples were centrifuged at 3000 rpm for 5 min. The supernatant pipetted out, and after adding UPW to the pellet in the tube, centrifugation was repeated three times. Then samples were dried at 30 °C. The powder-like samples were resolved by adding 20 µl of immersion oil and vortexed. The suspension was dropped on slides and the cover slips were fixed with clear nail polish. Micro-remains were identified and photographed under Nikon Eclipse polarizing light microscope at a magnification of 400x. The identification of the starch grains relied on several studies and our microparticle database^58–62^.

### Methods: macro-archaeobotanical analysis methods

The recovery of the macro-botanical remains followed the standard wet sieving process detailed by Kenward et al.^63^. Heavy and light fractions were separated and dried. Inorganic particles (e.g. pebbles, daub fragments etc.), and organic particles were separated. During preparation, the light fraction of the samples was processed under a stereo microscope. The items for macro-archaeobotanical examination, such as fruits, seeds, charcoal pieces, and stem and leaf fragments of the Poaceae family, were separated prior to identification. Sample preparation and identification were carried out with an Olympus SZX7 binocular stereomicroscope with an attached digital camera. Identification of the plant remains was performed using a modern reference collection of relevant taxa and the works of Schermann^64^ and Cappers et al.^65^. Weeds and other wild plants were grouped according to the applicable parameters in Borhidi’s relative ecological indicator system. In addition, the wild plant descriptions are based on Brombacher and Jacomet^66^. Scientific plant names of cultivars provided are listed in Zohary et al.^46^.

## Electronic supplementary material

Below is the link to the electronic supplementary material.

Supplementary Material 1

Supplementary Material 2

Supplementary Material 3

Supplementary Material 4

## Data Availability

All data generated or analysed during this study are included in this published article (and its supplementary information files).
