# Supplementary material for: Isotope and archaeobotanical analysis reveal radical changes in mobility, diet and inequalities around 1500 BCE at the core of Europe
Source: Sci Rep. 2025 May 20;15:17494. doi: 10.1038/s41598-025-01113-z (PMC12092678; doi:10.1038/s41598-025-01113-z)
Supplement: Supplementary file 1 — Supplementary Material 1 [file 41598_2025_1113_MOESM1_ESM.docx]

Supplementary Materials for

**Isotope and archaeobotanical analysis reveal radical changes in mobility, diet and inequalities around 1500 BCE at the core of Europe**

Cavazzuti *et al.*

*Corresponding authors. Email: Claudio Cavazzuti: claudio.cavazzuti3@unibo.it; Anikó Horváth: horvatha@atomki.hu; István Major: imajor@atomki.hu; Tamás Hajdu: tamas.hajdu@ttk.elte.hu

**This file includes:**

Supplementary Text

Figs. S1 to S3

Tables S1 to S4

References (1 to 20)

**Supplementary Text**

Archaeological contexts

**Tiszafüred-Majoroshalom**

Between 1961 and 1972 1006 burials were excavated at the Tiszafüred-Majoroshalom Bronze Age cemetery: 2 cremation graves of the Hatvan culture, 622 inhumation burials of the Otomani–Füzesabony culture, respectively 382 inhumation and cremation graves of the Tumulus culture (1–4). The preliminary archaeological analysis of the Tumulus culture cemetery (2) and the material of some outstanding graves of the Otomani–Füzesabony culture cemetery have already been published in several papers (4–10), however, the complete Middle Bronze Age cemetery has yet to be published.

The site was also later used as a burial place in the Avar period (from the mid-7th to the 9th century), as evidenced by the 1282 burials unearthed here (11).

**Gelej-Kanálisdűlő**

Two Middle Bronze Age cemetery sections were excavated at the sites Gelej-Kanális-dűlő and Gelej-Beltelek-dűlő. Hungarian research classifies these as Füzesabony culture sites, whereas international research considers them to be a part of the Otomani-Füzesabony Cultural Complex (OFCC) (12). The Middle Bronze Age burials were published by Tibor Kemenczei (13).12 burials were excavated in 1941, later in the Kanális-dűlő: 137 burials, Beltelek-dűlő: 21 burials, 170 burials in total. Anthropological analysis was carried out on 112 Middle Bronze Age burials, which was conducted by Tamás Hajdu and Ivett Kővári (3, 14). 12 Middle Bronze Age burials from Gelej-Kanális-dűlő and one burial from Gelej-Beltelek-dűlő were included in the research work of the Momentum Mobility Research Group and the National Research, Development and Innovation Office project no. 128013. The aim of the examination of these burials was to provide a more precise chronology for the cemetery and to perform a complex bio-archaeological analysis (including archaeological, anthropological, molecular biology and stable isotope studies) of the Middle Bronze Age communities that lived in the area of present-day Hungary. The burials used as samples were selected based on both archaeological and anthropological criteria. Current analysis of the burials suggests the distinction of three typological groups: one Classic and one Late Füzesabony style, as well as the Füzesabony-Koszider style. Out of the 13 burials examined, 7 were infant burials (two burials of children aged 1–2, and one by one burials of children aged 2–3, 3–4, 4–5, 8–9 and 9–10) and 6 were adult burials (three burials with mature male remains, three burials with mature female remains and one burial where biological sex could not be identified (15).

**Csanytelek-Palé**

92 MBA burials were excavated in 1988: 49 inhumations, 34 urn burials, 9 scattered cremation burials with the vessels of the Vatya, Maros and the Transdanubian Encrusted Pottery. However, grave furniture was mostly employed according to the Vatya burial practice, the different pottery types and the presence of various burial rites, the possible infiltration/movement of small groups from Transdanubia to this region cannot be ruled out as a consequence of intense exchange activities (16–18).

**Tiszapalkonya Erőmű**

The cemetery of the Füzesabony culture was excavated by Nándor Kalicz with 7 burials, after a formerly discovered and disturbed burial in 1958 (grave nr. 8; (6)). Burials can be dated to MBA 3, Koszider period (19).

**Rákóczifalva-Kastélydomb**

In 1962, a biritual Early and Late Bronze Age cemetery (cremation and inhumation burials) were excavated by Zsolt Csalog at Rákóczifalva-Kastélydomb (Jász-Nagykun-Szolnok County, Hungary). The 40 Late Bronze Age individuals from 37 burials were the bearers of the so-called Rákóczifalva cultural group of the Tumulus culture (20). Both the skeletal (18 individuals) and the cremated (24 individuals) remains were poorly preserved and fragmented. The age distribution of the Late Bronze Age community shows a high percentage of sub-adults (17 indiv., 40%) in the cemetery. The sex distribution was balanced (4 males, 6 females). The pathological alterations that are usually frequent in almost every prehistoric material were observable in this series too (e.g. degenerative alterations of the spine and joints, porotic hyperostosis and entheseal changes.

**Figure S1.** Geological map of the Central Hungary, ^87^Sr/^86^Sr environmental samples used as baselines, and location of the analysed sites. The geological map is constructed by using public domain wms data downloadable from <https://certmapper.cr.usgs.gov/data/apps/world-maps/>, which are of Public Domain. Credit: U.S. Geological Survey, Department of the Interior/USGS, U.S. Geological Survey.


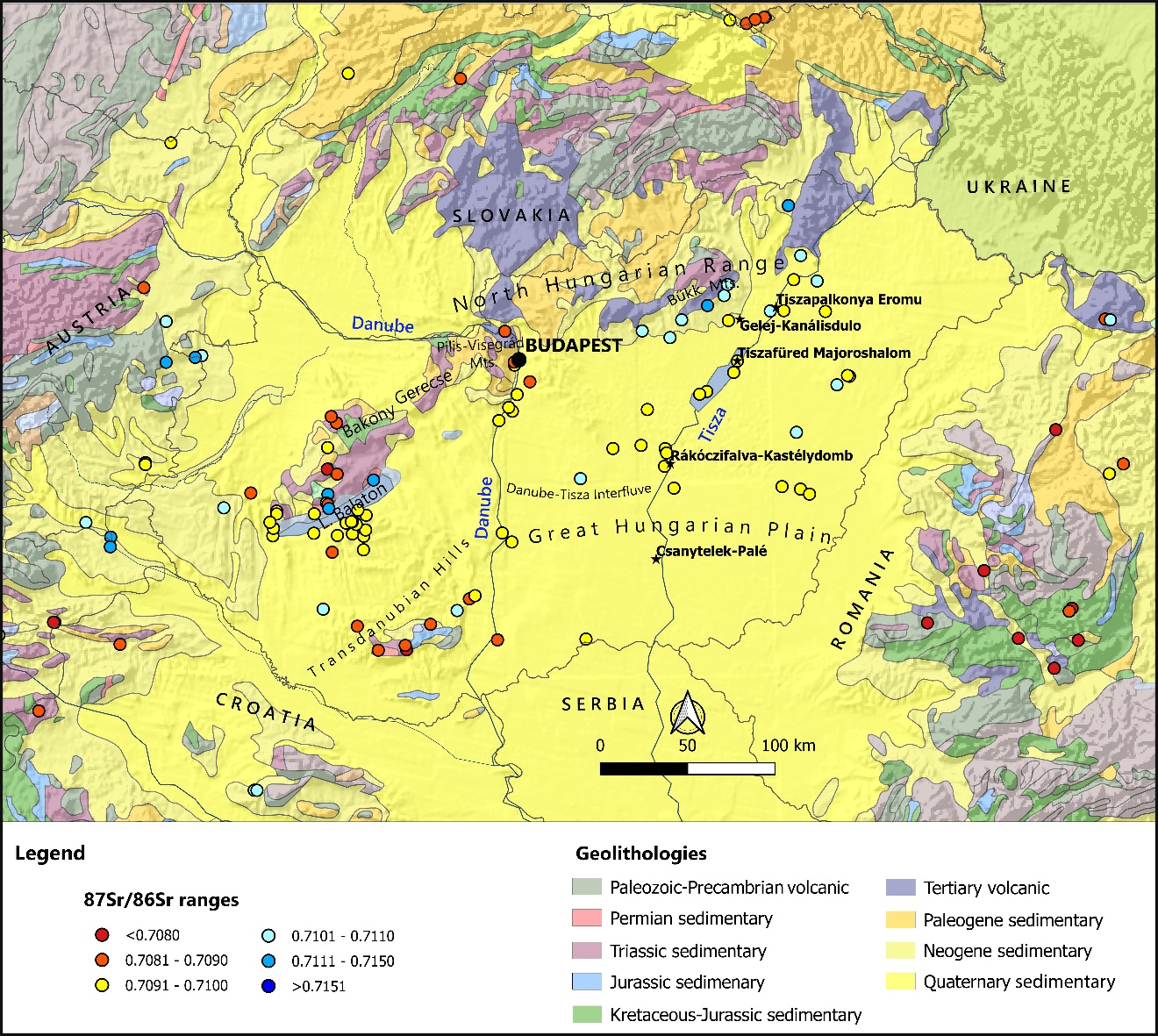


**Figure S2.** Distribution of ^87^Sr/^86^Sr values in the five analysed sites. TPR: Tiszapalkonya-Erőmű; GLK: Gelej-Kanálisdűlő; TFM: Tiszafüred-Majoroshalom; RKK: Rákóczifalva-Kastélydomb; CSP: Csanytelek-Palé.


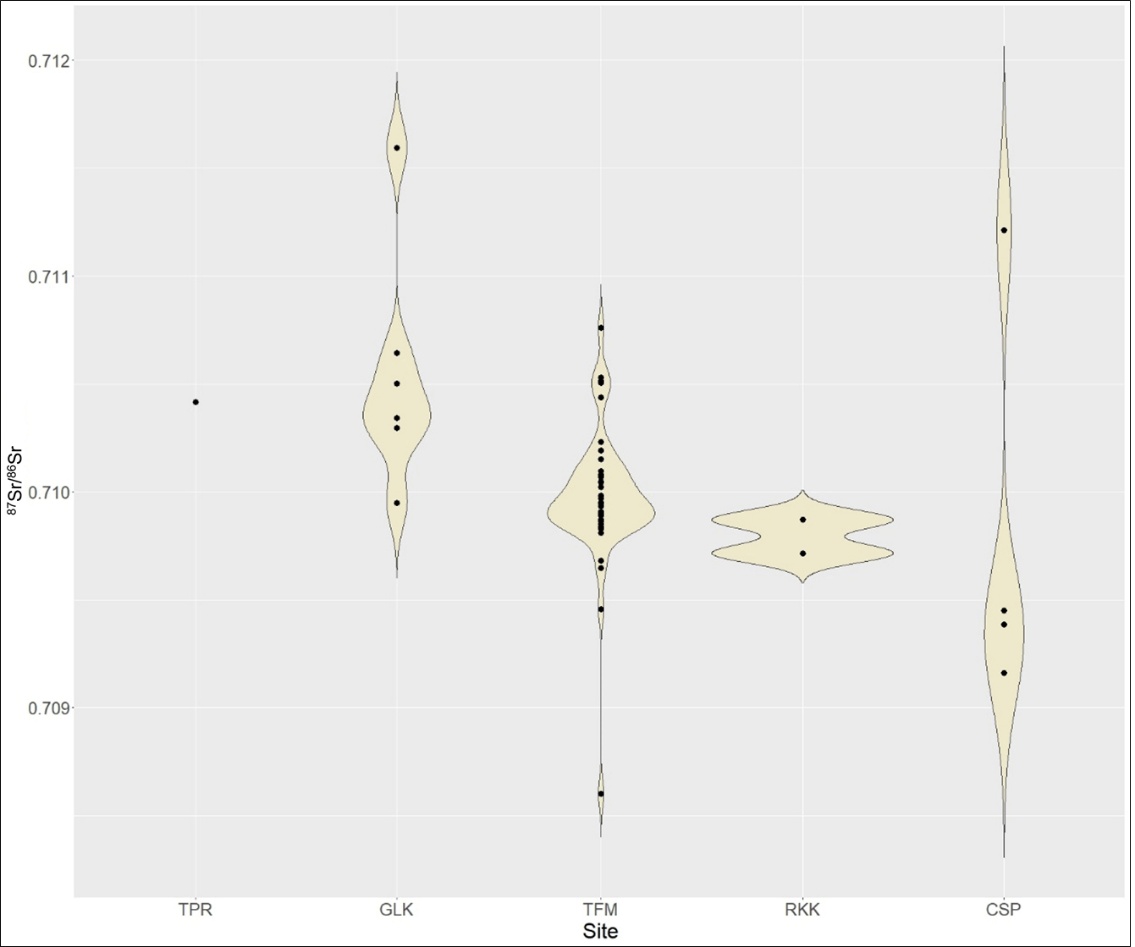


**Figure S3.** ^87^Sr/^86^Sr baselines from Tiszafüred-Majoroshalom area. The buffers around the site indicate territories at 5 km, 20 km and 50 km radius from the site.


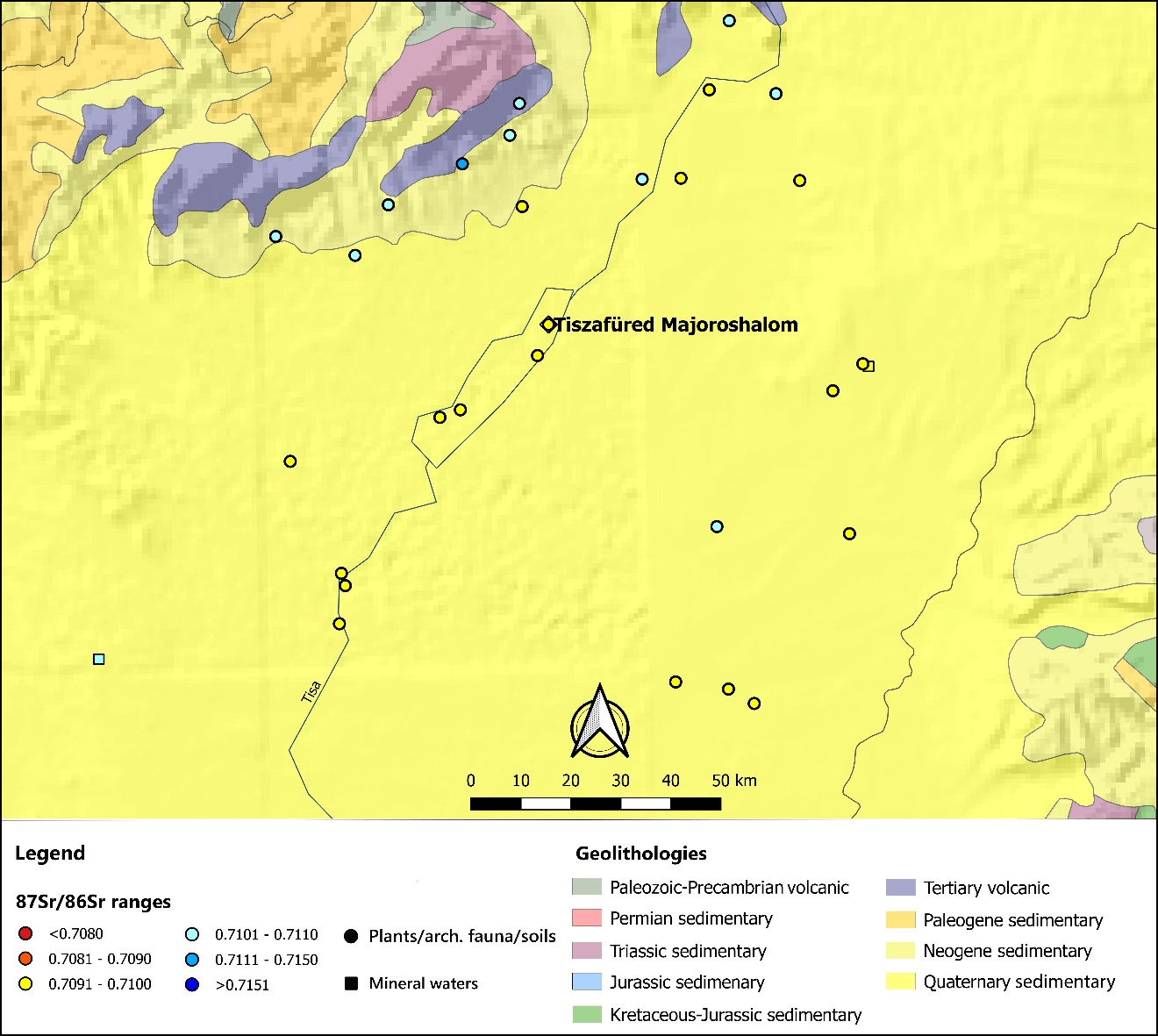


**Table S1.** List of archaeological contexts and samples for isotope analysis.

| **Site** | **Chronology** | **Culture** | **C and N samples** | **Sr samples** |
| --- | --- | --- | --- | --- |
| Tiszafüred Majoroshalom | MBA | Füzesabony | 31 | 33 |
|  | LBA | Tumulus | 13 | 13 |
| Tiszapalkonya Erőmű | MBA | Füzesabony | 1 | 1 |
| Gelej-Kanálisdűlő | MBA | Füzesabony | 7 | 7 |
| Csanytelek-Palé | MBA | Vatya | 4 | 4 |
| Rákóczifalva-Kastélydomb | LBA | Tumulus | 2 | 2 |
| Total human |  |  | 58 | 60 |

**Table S2.** List of human samples and results

| **Site** | **Grave number** | **Phase** | **Osteological sex** | **Age category (Age years)** | **AMS 14C measuring id** | **^14^C date**  **(BP)** | **Calibrated**  **^14^C date**  **(years BCE)** | **Sample for ^87^Sr/^86^Sr** | **^87^Sr/^86^Sr** | **± 1σ** | **Sample for ^14^C, δ^15^N**  **and δ^13^C** | **δ^15^N sample/ AIR** | **N:W%** | **δ^13^C sample/PDB** | **C:W%** | **C/N** |
| --- | --- | --- | --- | --- | --- | --- | --- | --- | --- | --- | --- | --- | --- | --- | --- | --- |
| TFM | B12 | MBA | M | 30-39 | DeA-36138 | 3270±18 | 1620-1460 | LM_1_ | 0.710761 | 0.000023 | cranial fr. | 11.3 | 13.1 | -19.4 | 37.3 | 3.3 |
| TFM | B54 | MBA | M | 20-29 | DeA-31087 | 3429±34 | 1880-1620 | LM_1_ | 0.709945 | 0.000020 | vertebra | 12.2 | 14.4 | -19.1 | 42.2 | 3.4 |
| TFM | B69 | MBA | M | 35-44 | DeA-36283 | 3282±22 | 1620-1500 | LM_1_ | 0.710068 | 0.000022 | cranial fr. | 11.8 | 13.5 | -20.2 | 35.5 | 3.1 |
| TFM | B112 | MBA | ? | 2-4 | DeA-36150 | 3329±20 | 1670-1520 | RM^1^ | 0.709810 | 0.000027 | cranial fr. | 11.5 | 7.9 | -19.1 | 25 | 3.7 |
| TFM | B114 | MBA | M | 30-35 | DeA-36133 | 3421±20 | 1870-1850; 1770-1630 | LM_1_ | 0.709971 | 0.000021 | rib | 11.4 | 14.6 | -19.3 | 39.8 | 3.2 |
| TFM | B115 | MBA | M | 30-39 | DeA-31088 | 3422±33 | 1880-1620 | RM^2^ | 0.709901 | 0.000025 | rib | 10.9 | 15.5 | -19.2 | 40.1 | 3 |
| TFM | B136 | MBA | M | 40-44 | DeA-36156 | 3431±22 | 1880-1630 | RM_1_ | 0.710233 | 0.000020 | rib | 11.9 | 11.7 | -19.5 | 33.3 | 3.3 |
| TFM | B146 | MBA | ? | 3-4 | DeA-36135 | 3385±19 | 1740-1620 | RM^1^ | 0.709903 | 0.000021 | petrous | 11.3 | 15.2 | -19.2 | 41.7 | 3.2 |
| TFM | B159 | MBA | ? | 16-18 | DeA-42311 | 3347±29 | 1880-1620 | LM_1_ | 0.709837 | 0.000022 | metacarpal | 10.6 | 12.3 | -20.3 | 32.3 | 3.1 |
| TFM | D19 | MBA | ? | 9-10 | DeA-36434 | 3334±22 | 1690-1530 | LM_1_ | 0.709953 | 0.000022 | cranial fr. | 10.6 | 14.9 | -19.5 | 39.6 | 3.1 |
| TFM | D34 | MBA | F | 25-29 | DeA-36148 | 3357±20 | 1740-1540 | RM_1_ | 0.710505 | 0.000027 | rib | 9.6 | 12 | -19.6 | 35.9 | 3.5 |
| TFM | D37 | MBA | M | 25-35 | DeA-36273 | 3352±23 | 1740-1540 | RM_1_ | 0.710514 | 0.000027 | cranial fr. | 11.3 | 15.9 | -19.6 | 41.3 | 3 |
| TFM | D38 | MBA | F | 30-39 | - | - | - | RM^1^ | 0.710047 | 0.000024 | - | - | - | - | - | - |
| TFM | D41 | MBA | M | 35-49 | DeA-36271 | 3326±21 | 1670-1510 | LM2 | 0.710190 | 0.000023 | rib | 10.9 | 15.5 | -19.8 | 40.9 | 3.1 |
| TFM | D48 | MBA | M | 15-18 | DeA-36278 | 3420±23 | 1870-1850; 1770-1620 | LM^2^ | 0.709892 | 0.000025 | sphenoid | 11 | 14.7 | -20.1 | 39.3 | 3.1 |
| TFM | D49 | MBA | ? | 12,5-13,5 | DeA-36267 | 3299±21 | 1620-1510 | RM1 | 0.709872 | 0.000017 | cranial fr. | 10.2 | 14.4 | -19.9 | 41 | 3.3 |
| TFM | D56 | MBA | M | 45-54 | DeA-31089 | 3368±34 | 1750-1530 | LC1 | 0.710096 | 0.000022 | cranial fr. | 11.4 | 13.1 | -19.9 | 37.4 | 3.3 |
| TFM | D76 | MBA | M | 50-59 | DeA-36284 | 3462±23 | 1880-1690 | LC_1_ | 0.710078 | 0.000023 | rib | 11.4 | 14.8 | -19.7 | 38.1 | 3 |
| TFM | D77 | MBA | F | 20-24 | DeA-36269 | 3388±21 | 1750-1620 | LM^1^ | 0.709865 | 0.000023 | rib | 9.7 | 14.2 | -19.5 | 38.6 | 3.2 |
| TFM | D104 | MBA | M | 30-39 | DeA-36140 | 3341±18 | 1730-1530 | LC1 | 0.710531 | 0.000023 | mandible | 11.3 | 14.8 | -19.4 | 42.4 | 3.4 |
| TFM | D106 | MBA | ? | 8-10 | DeA-33615 | 3355±21 | 1740-1540 | RM_1_ | 0.710152 | 0.000020 | sphenoid | 9.8 | 12.2 | -20 | 35.5 | 3.4 |
| TFM | D113 | MBA | F | 50-59 | DeA-36281 | 3285±22 | 1620-1500 | LM_1_ | 0.710095 | 0.000019 | cranial fr. | 11 | 14.7 | -19.8 | 38.5 | 3.1 |
| TFM | D117 | MBA | M | 20-39 | DeA-36274 | 3368±22 | 1740-1540 | LM_1_ | 0.710021 | 0.000024 | sphenoid | 11.6 | 14.7 | -20.3 | 39.1 | 3.1 |
| TFM | D123 | MBA | F | 35-39 | DeA-36147 | 3359±19 | 1740-1540 | RM_1_ | 0.709647 | 0.000025 | rib | 10.7 | 14.6 | -19.6 | 42.4 | 3.4 |
| TFM | D136 | MBA | M | 40-59 | DeA-36268 | 3375±21 | 1750-1560 | LM2 | 0.709680 | 0.000027 | dentine | 10.8 | 14.6 | -19.5 | 40.9 | 3.3 |
| TFM | D155 | MBA | ? | 14-16 | DeA-31483 | 3412±28 | 1870-1850; 1770-1620 | LM_1_ | 0.709909 | 0.000021 | sphenoid | 11 | 13.2 | -20.5 | 38.1 | 3.4 |
| TFM | D240 | MBA | M | 25-29 | DeA-42310 | 3422±31 | 1740-1530 | M_1_ | 0.710437 | 0.000023 | rib | 12 | 14.4 | -20.6 | 36.7 | 3.0 |
| TFM | D267 | MBA | ? | 3-4 | DeA-31484 | 3346±29 | 1740-1530 | RM^1^ | 0.709890 | 0.000019 | rib | 12.5 | 14 | -20.1 | 40.4 | 3.4 |
| TFM | D284 | MBA | M | 35-49 | DeA-36136 | 3319±19 | 1630-1520 | RC1 | 0.710150 | 0.000024 | mandible | 11.4 | 11 | -19.2 | 31.3 | 3.3 |
| TFM | D285 | MBA | ? | 18-19 | DeA-33614 | 3313±21 | 1630-1510 | LM1 | 0.709969 | 0.000023 | parietal | 9.3 | 10.5 | -19.9 | 29.1 | 3.2 |

**Table S2.** List of human samples and results (continued)

| **Site** | **Grave number** | **Phase** | **Osteological sex** | **Age years** | **AMS ^14^C measuring id** | **^14^C date**  **(BP)** | **Calibrated**  **^14^C date**  **(years BCE)** | **Sample for ^87^Sr/^86^Sr** | **^87^Sr/^86^Sr** | **± 1σ** | **Sample for ^14^C, δ15N and δ13C** | **δ^15^N sample/ AIR** | **N:W%** | **δ^13^C sample/PDB** | **C:W%** | **C/N** |
| --- | --- | --- | --- | --- | --- | --- | --- | --- | --- | --- | --- | --- | --- | --- | --- | --- |
| TFM | D287 | MBA | ? | Neonate | - | - | - | RM1 | 0.709868 | 0.000023 | - | - | - | - | - | - |
| TFM | D304 | MBA | ? | 15-19 | DeA-31485 | 3426±29 | 1880-1620 | LM1 | 0.709850 | 0.000023 | rib | 9.8 | 13 | -19.5 | 41.8 | 3.8 |
| TFM | D305 | MBA | ? | 8-9 | DeA-36265 | 3433±23 | 1880-1630 | LM1 | 0.709982 | 0.000024 | rib | 9.6 | 14 | -19.4 | 39.3 | 3.3 |
| TFM | E10 | LBA | M | 40-55 | DeA-36154 | 3137±19 | 1500-1310 | RM1 | 0.709895 | 0.000025 | vertebra | 11 | 13.6 | -16.8 | 41.9 | 3.6 |
| TFM | E16 | LBA | M | 45-49 | DeA-33619 | 3147±20 | 1500-1320 | RM1 | 0.708601 | 0.000025 | rib | 11.4 | 14.8 | -17.3 | 41.8 | 3.3 |
| TFM | E24 | LBA | M | 30-34 | DeA-36130 | 3128±20 | 1450-1300 | RM1 | 0.709861 | 0.000019 | sphenoid | 10.9 | 14.9 | -16.6 | 42 | 3.3 |
| TFM | E31 | LBA | ? | 5-6 | DeA-36134 | 3115±19 | 1440-1300 | RM1 | 0.709949 | 0.000023 | rib | 10.9 | 15.7 | -18.1 | 43.3 | 3.2 |
| TFM | E62 | LBA | M | 25-29 | DeA-36145 | 3068±18 | 1410-1270 | RM1 | 0.709982 | 0.000017 | rib | 10.4 | 13.9 | -17.1 | 40.6 | 3.4 |
| TFM | C107 | LBA | F | 25-29 | DeA-33617 | 3224±20 | 1530-1430 | RM1 | 0.709834 | 0.000025 | rib | 10.5 | 14 | -18.5 | 39.5 | 3.3 |
| TFM | C154 | LBA | M | 25-35 | DeA-36151 | 3211±19 | 1510-1430 | LM2 | 0.709455 | 0.000021 | metatarsal | 10.6 | 14 | -19.9 | 41.2 | 3.4 |
| TFM | C165 | LBA | M | 30-39 | DeA-36270 | 3186±21 | 1500-1420 | LM1 | 0.709863 | 0.000021 | metatarsal | 10.8 | 13.5 | -18 | 36.2 | 3.1 |
| TFM | C173 | LBA | F | 18-19 | DeA-36266 | 3011±21 | 1390-1130 | LM1 | 0.709894 | 0.000023 | rib | 10.1 | 14.2 | -16.9 | 40 | 3.3 |
| TFM | C174 | LBA | F? | 25-29 | DeA-36272 | 3076±21 | 1420-1270 | RM1 | 0.710080 | 0.000023 | sphenoid | 10.6 | 15.4 | -17.3 | 42 | 3.2 |
| TFM | C191 | LBA | M | 40-44 | DeA-36275 | 3140±21 | 1500-1310 | LM1 | 0.709934 | 0.000022 | rib | 10.2 | 15.9 | -18 | 40.8 | 3 |
| TFM | C265 | LBA | M | 30-34 | DeA-31487 | 3167±29 | 1510-1390; 1340-1320 | RC1 | 0.710045 | 0.000021 | sphenoid | 10.6 | 14.7 | -15.9 | 38.5 | 3 |
| TFM | C281 | LBA | ? | 16-19 | DeA-33618 | 3072±20 | 1410-1270 | LM1 | 0.709828 | 0.000025 | scapula | 10.8 | 13.1 | -15 | 35.1 | 3.1 |
| GLK | 106 | MBA | M | 50-59 | DeA-36131 | 3321±18 | 1630-1530 | LM1 | 0.710295 | 0.000035 | rib | 10.4 | 14.5 | -19.6 | 40.8 | 3.3 |
| GLK | 273 | MBA | M | 30-44 | DeA-36132 | 3350±19 | 1740-1540 | RM1 | 0.711595 | 0.000019 | vertebra | 12.1 | 15 | -19.9 | 40.5 | 3.2 |
| GLK | 124 | MBA | F | 45-54 | DeA-33620 | 3403±20 | 1750-1620 | LM1 | 0.710503 | 0.000201 | rib | 9.7 | 14.8 | -19.5 | 43.3 | 3.4 |
| GLK | 98 | MBA | M | 30-49 | DeA-36279 | 3306±21 | 1620-1510 | RC1 | 0.710342 | 0.000030 | cranial fr. | 9.8 | 13.7 | -19.5 | 36.9 | 3.2 |
| GLK | 15 | MBA | M | 35-44 | DeA-36139 | 3359±19 | 1740-1540 | LM1 | 0.70995 | 0.000027 | cranial fr. | 10.5 | 13.8 | -19.7 | 40 | 3.4 |
| GLK | 26 | MBA | F | 23-24 | DeA-36153 | 3386±19 | 1740-1620 | RM1 | 0.710343 | 0.000031 | rib | 10.1 | 13.2 | -19.8 | 39.7 | 3.5 |
| GLK | 227 | MBA | F | 50-59 | DeA-36155 | 3310±20 | 1620-1510 | RM1 | 0.710645 | 0.000019 | cranial fr. | 11.4 | 13.7 | -20.1 | 42.5 | 3.6 |
| CSP | 73 | MBA | M | 50+ | DeA-36146 | 3391±19 | 1750-1620 | LM1 | 0.711211 | 0.000034 | sphenoid | 10.1 | 13.6 | -19.5 | 40.3 | 3.5 |
| CSP | 34 | MBA | M | 20-39 | DeA-36280 | 3376±22 | 1750-1610; 1580-1560 | RM1 | 0.709161 | 0.000033 | cranial fr. | 11.1 | 14.9 | -19.8 | 37.9 | 3 |
| CSP | 27 | MBA | F | 20-59 | DeA-36149 | 3358±20 | 1740-1540 | LM2 | 0.709449 | 0.000040 | vertebra | 10.5 | 13.7 | -20 | 41.1 | 3.5 |
| CSP | 72 | MBA | F | 20-39 | DeA-36152 | 3391±20 | 1750-1620 | RM1 | 0.709387 | 0.000029 | rib | 10.4 | 13.8 | -20.3 | 41.6 | 3.5 |
| TPR | 2 | MBA | F? | 22-24 | DeA-36282 | 3412±22 | 1870-1620 | LM1 | 0.710417 | 0.000021 | rib | 11.1 | 15.2 | -20 | 39.7 | 3 |
| RKK | 155 | LBA | M | 23-39 | DeA-36143 | 3063±18 | 1410-1260 | RM1 | 0.709716 | 0.000023 | rib | 9.6 | 12.1 | -16.3 | 34.9 | 3.4 |
| RKK | 163 | LBA | F | 23-39 | DeA-36144 | 3095±19 | 1430-1290 | RM1 | 0.709871 | 0.000023 | rib | 10 | 14.7 | -16.9 | 42 | 3.3 |

**Table S3.** List of animal and environmental samples.

| **Site** | **Type of sample** | **Sample** | **Phase** | **Sample for ^87^Sr/^86^Sr** | **^87^Sr/^86^Sr** | **± 1σ** | **Sample for ^14^C, δ^15^N and δ^13^C** | **δ^15^N sample/AIR** | **N:W%** | **δ^13^C sample/PDB** | **C:W%** | **C/N** |
| --- | --- | --- | --- | --- | --- | --- | --- | --- | --- | --- | --- | --- |
| TFM III | animal | horse - first molar enamel | MBA | horse - first molar enamel | 0.709814 | 0.000020 | - | - | - | - | - | - |
| TFM III | animal | horse - second molar enamel | MBA | horse - second molar enamel | 0.709773 | 0.000023 | - | - | - | - | - | - |
| TFM III | animal | horse - third molar enamel | MBA | horse - third molar enamel | 0.709798 | 0.000026 | - | - | - | - | - | - |
| TFM III | animal | cattle - molar enamel | MBA | cattle - molar enamel | 0.709778 | 0.000022 | - | - | - | - | - | - |
| TFM III | animal | goat/sheep - molar enamel | MBA | goat/sheep - molar enamel | 0.710735 | 0.000035 | - | - | - | - | - | - |
| TFM III | animal | pig - molar enamel | MBA | pig - molar enamel | 0.709887 | 0.000034 | - | - | - | - | - | - |
| TFM III | animal | dog - molar enamel | MBA | dog - molar enamel | 0.709962 | 0.000039 | cervical vertebra | 11,7 | 10,9 | -18,9 | 30,9 | 3,3 |
| TFM III | animal | pig | MBA | - | - | - | pig- scapula | 10,4 | 13,2 | -22,2 | 35,5 | 3,1 |
| TFM III | animal | cattle | MBA | - | - | - | cattle - cranial fragment | 7,8 | 8,2 | -20,1 | 23,5 | 3,3 |
| TFM III | animal | pig | MBA | - | - | - | pig - fragment of maxilla | 9,3 | 10,7 | -20,6 | 29,8 | 3,2 |
| TFM III | animal | pig | MBA | - | - | - | pig - rib | 13,1 | 10,6 | -21,4 | 30,7 | 3,4 |
| TFM III | animal | pig | MBA | - | - | - | pig - rib | 12,9 | 12 | -21,7 | 34,1 | 3,3 |
| TFM | environmental | soil sample (depth 120 cm) |  | soil sample (depth 120 cm) | 0.710101 | 0.000024 | - | - | - | - | - | - |
| TFM | environmental | rodent bone | - | rodent bone | 0.709920 | 0.000022 | - | - | - | - | - | - |
| TFM | environmental | plant remains from a skull |  | plant remains from a skull | 0.709926 | 0.000021 | - | - | - | - | - | - |
| TFM | environmental | shell | - | shell | 0.709633 | 0.000024 | - | - | - | - | - | - |

**Table S4.** Descriptive statistics of δ^13^C and δ^15^N (‰) in the two macro-phases (*animals are not radiocarbon dated, but they were collected from Tiszafüred Majoroshalom settlement, which is abandoned at the end of the MBA)).

| **Sample** | **Isotope ratio** | **MBA** | | | | **LBA** | | | |
| --- | --- | --- | --- | --- | --- | --- | --- | --- | --- |
|  |  | Mean | Std. Dev. | Min. | Max | Mean | Std. Dev. | Min. | Max. |
| Humans | δ^13^C | -19.7 | 0.3 | -20.5 | -19.1 | -17.2 | 1.2 | -19.9 | -15.0 |
|  | δ^15^N | 10.8 | 0.8 | 9.3 | 12.5 | 10.6 | 0.5 | 9.6 | 11.4 |
| Animals* | δ^13^C | -20.8 | 1.2 | -22.2 | -18.9 |  |  |  |  |
|  | δ^15^N | 10.9 | 2.1 | 7.8 | 13.1 |  |  |  |  |

**References**

1. Csalog, Z. Tiszafüred-Majoroshalom (Kom. Szolnok. Kr. Tiszafüred). *Archaeol Ért* **89**. 259 (1962).

2. Kovács, T. *Tumulus Culture cemeteries of Tiszafüred* (Magyar Nemzeti Múzeum. 1975).

3. Hajdu, T. *A bronzkori Füzesabony- és Halomsíros kultúra népességének biológiai rekonstrukciója*. PhD Disssertation (Eötvös Loránd University, Budapest 2012).

4. J. Dani. et al. New radiocarbon dates from the Bronze Age Tiszafüred-Majoroshalom site (Eastern Hungary). *Radiocarbon* 1–13. https://doi.org/10.1017/RDC.2024.123 (2024).

5. Kovács T. Korai markolatlapos bronztőrök a Kárpát-medencében. [Frühe Bronzedolche mit Griffplatte im Karpatenbecken]. *Archaeol. Ért* **100**. 157–166 (1973).

6. Kovács, T. Középső bronzkori aranyleletek Magyarországról [Mittelbronzezeitliche Goldfunde aus Nordost-Ungarn]. *Folia Archaeol.* **30**. 55–75 (1979).

7. Kovács T. Einige neue Angaben zur Ausbildung und inneren Gliederung der Füzesabony-Kultur in *Südosteuropazwischen 1600 Und 1000 v.Chr. Prähistorische Archäologie in Südosteuropa 1*. (ed. Hänsel, B.) 287–307 (Moreland. 1982).

8. Kovács T. Füzesabony-Kultur in *Kulturen Der Frühbronzezeit Des Karpatenbeckens Und Nordbalkans. Balcano-Pannonica. Sonderausgabe 22*. (ed. Tasić N.) 235–255 (Balkanološki Institut SANU. 1984).

9. Kovács, T. Bestattungssitten der Füzesabony-Kultur und das Gräberfeld von Tiszafüred-Majoroshalom in *Bronzezeit in Ungarn. Forschungen in Tell-Siedlungenan Donau Und Theiss*. (ed. Meier-Arendt, W.) 98–98 (Museum für Vor- und Frühgeschichte –Archäologisches Museum. Pytheas. 1992).

10. Kovács, T. Die terminologischen und chronologischen Probleme der frühen und mittleren Bronzezeit in Ostungarn in *Il Passaggio Dal Neolitico All’età Del Bronzo Nell’europa Centrale e Nella Regione Alpina. Problemi Cronologici e Terminologici. Atti Del X Simp. Int. Neol. Età Bronzo in Europa. Lazise-Verona 8-12 Aprile 1980 (Verona 1982)*. (ed. Aspes, A.) 153–164 (Museo Civico di Storia Naturale. 1982).

11. Garam, É. *Das awarenzeitliche Gräberfeld in Tiszafüred. Cemeteries of the Avar Period /567-829/ in Hungary.* (Akadémiai Kiadó. 1995).

12. P. Fischl, K. Settlement layouts. systems and structure of the Otomani-Füzesabony Cultural Complex. *Gesta* **17**. 3–8 (2018).

13. Kemenczei, T. *Das mittelbronzezeitliche Gräberfeld von Gelej* (Magyar Nemzeti Múzeum. 1979).

14. Kővári, I. Az Alföld őskori népességeinek megítélése kraniometriai elemzésük révén. PhD Dissertation (University of Debrecen 2008).

15. Kiss, V. et al. Middle Bronze Age cemeteries in Gelej in *Universitätsforschungen Zur Prähistorischen Archäologie*. pp. 65–85. (Habelt Verlag 2023).

16. Kiss V. *Middle Bronze Age Encrusted Pottery in Western Hungary* (Archaeolingua, Budapest 2012).

17. Lőrinczy, G., Trogmayer, O. Birituális vatyai temető Csanytelek-Palén in *A Móra Ferenc Múzeum Évkönyve: Studia Archaeologica 1* (1995), 49–90.

18. Szalai, F. A Csanytelek-Palén feltárt középső bronzkori csontvázleletek antropológiai vizsgálata in *A Móra Ferenc Múzeum Évkönyvei: Studia Archaeologica 1* (1995). 91–121.

19. Jaeger, M. et al.. Baltic Amber in the Hungarian Bronze Age. New data and current stage of research. *Spraw. Archeol.* **75**. 138–186 (2023).

20. Hajdu, T. A Rákóczifalva-Kastélydombon feltárt bronzkori embertani leletek vizsgálatának eredményei. *Anthropol. Kozl.* **61**. 3–24 (2020).
