# Supplementary figures and images for: Isotope and archaeobotanical analysis reveal radical changes in mobility, diet and inequalities around 1500 BCE at the core of Europe
Source: Sci Rep. 2025 May 20;15:17494. doi: 10.1038/s41598-025-01113-z (PMC12092678; doi:10.1038/s41598-025-01113-z)

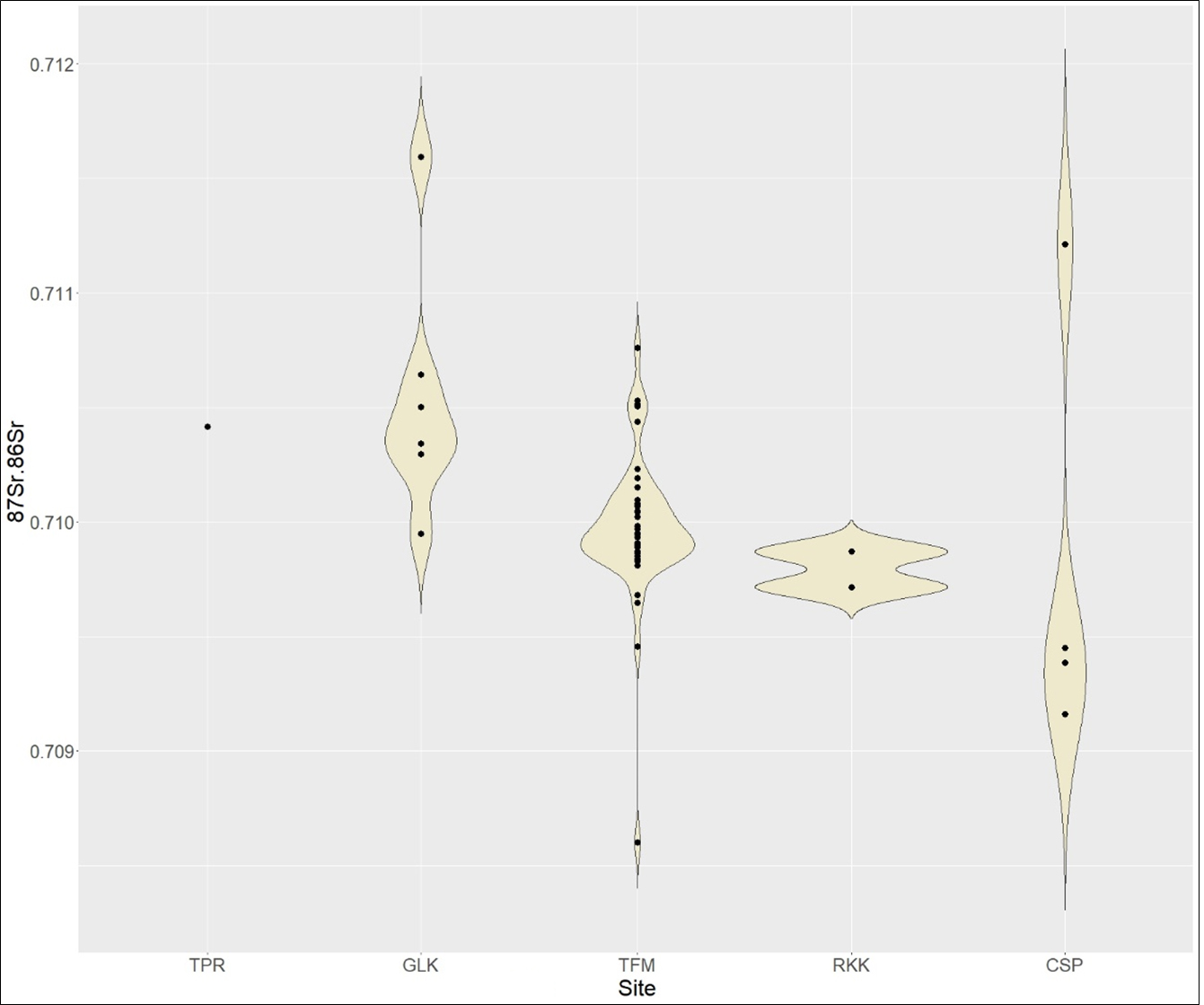

Supplement: Supplementary file 2 — Supplementary Material 2 [file 41598_2025_1113_MOESM2_ESM.jpg]

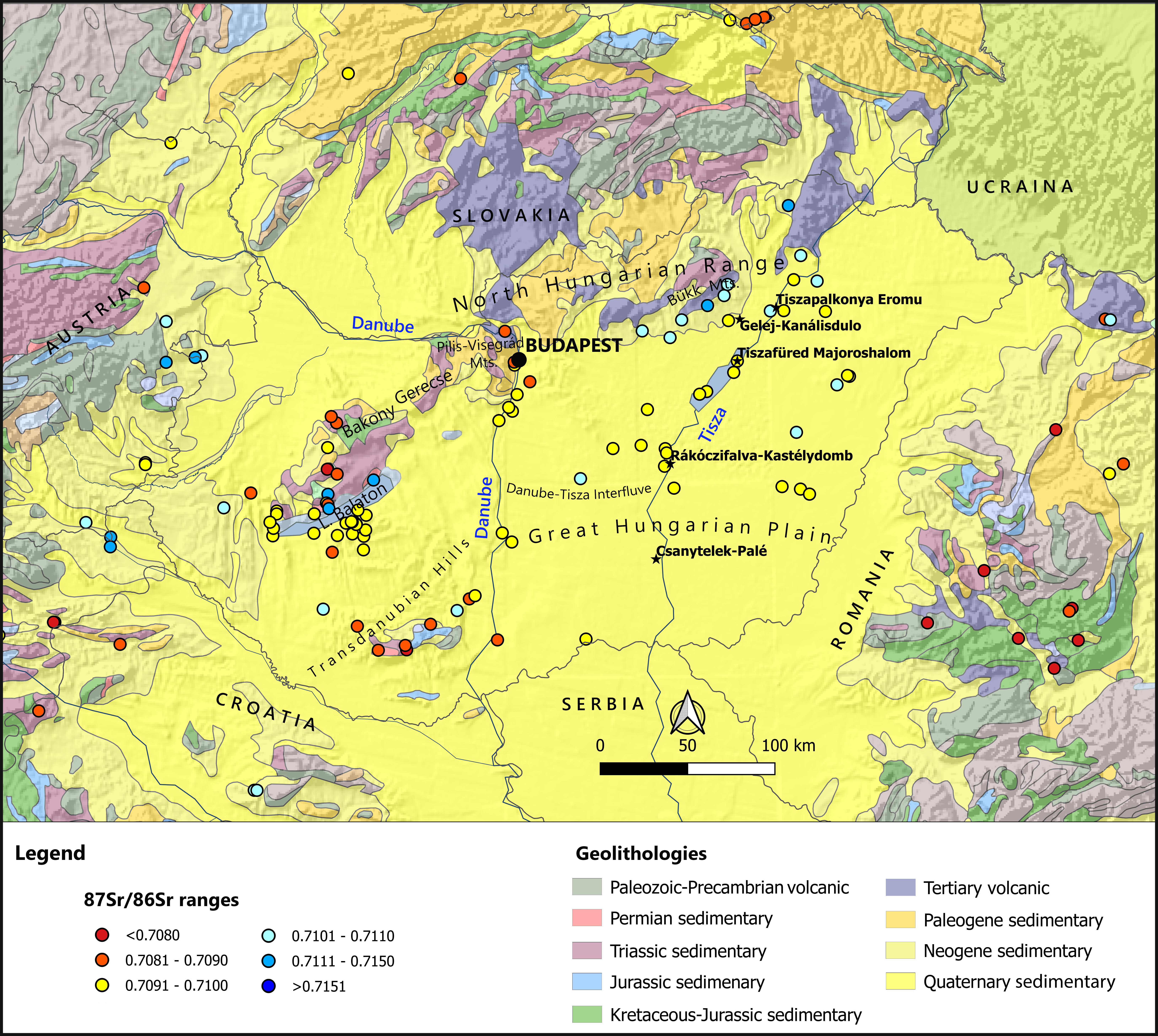

Supplement: Supplementary file 3 — Supplementary Material 3 [file 41598_2025_1113_MOESM3_ESM.jpg]

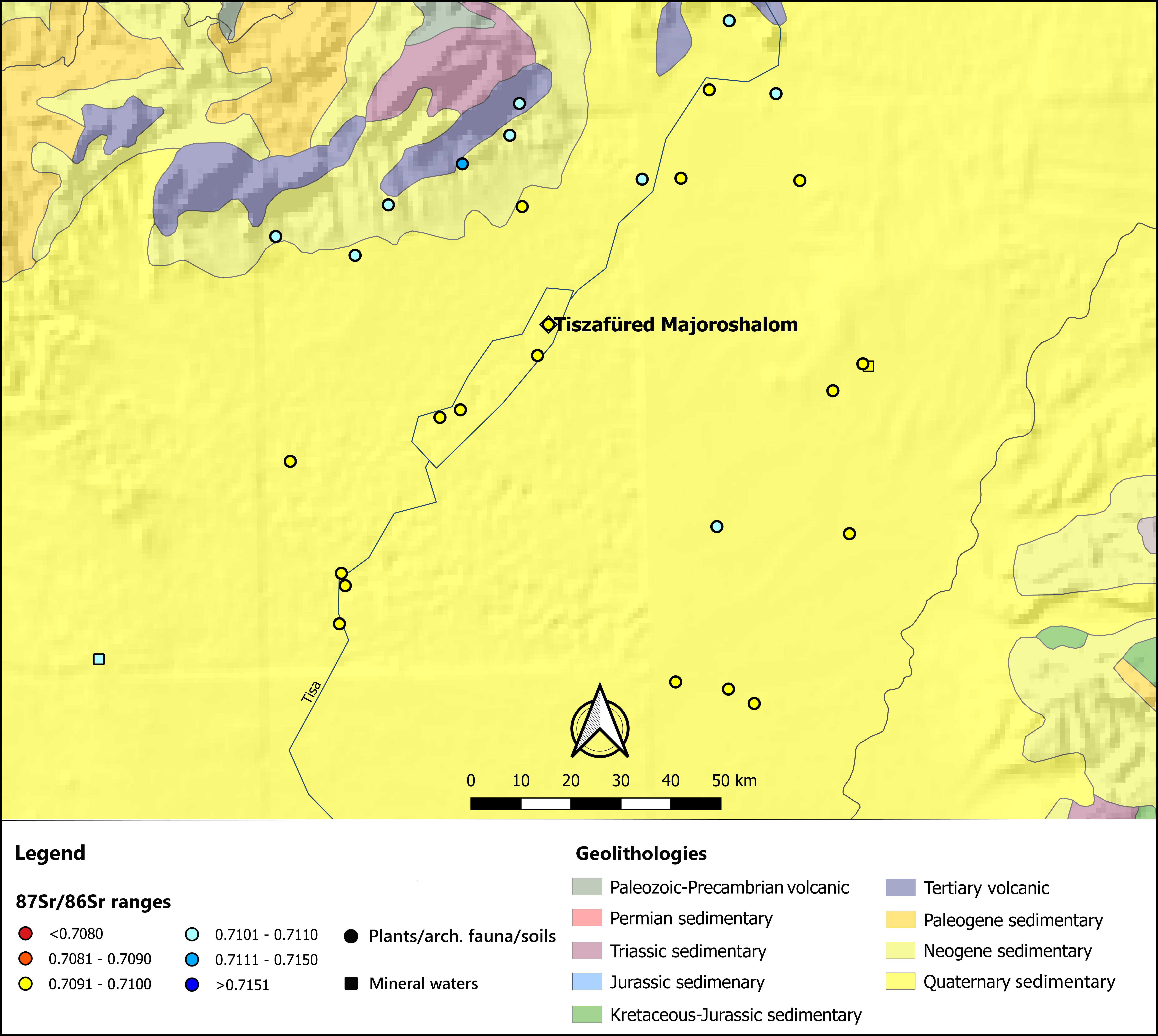

Supplement: Supplementary file 4 — Supplementary Material 4 [file 41598_2025_1113_MOESM4_ESM.jpg]
